# Integrative bioinformatics analysis of WDHD1: a potential biomarker for pan-cancer prognosis, diagnosis, and immunotherapy

**DOI:** 10.1186/s12957-023-03187-3

**Published:** 2023-09-27

**Authors:** Zhiwei Cui, Fan Zou, Rongli Wang, Lijun Wang, Feiyan Cheng, Lihui Wang, Rumeng Pan, Xin Guan, Nini Zheng, Wei Wang

**Affiliations:** 1 Department of Obstetrics and Gynecology, The First Affiliated Hospital of Xi’an Jiaotong University, Xi’an, China; 2 Department of Respiratory and Critical Care Medicine, Affiliated Hospital of Zunyi Medical University, Zunyi, China; 3 Department of Anesthesiology, The First Affiliated Hospital of Xi’an Jiaotong University, No. 277, Yanta West Road, Xi’an, 710061 Shaanxi China

**Keywords:** WDHD1, Pan-cancer, Prognosis, Diagnosis, Tumor immunity

## Abstract

**Background:**

Although WD repeat and high-mobility group box DNA binding protein 1 (WDHD1) played an essential role in DNA replication, chromosome stability, and DNA damage repair, the panoramic picture of WDHD1 in human tumors remains unclear. Hence, this study aims to comprehensively characterize WDHD1 across 33 human cancers.

**Methods:**

Based on publicly available databases such as TCGA, GTEx, and HPA, we used a bioinformatics approach to systematically explore the genomic features and biological functions of WDHD1 in pan-cancer.

**Results:**

WDHD1 mRNA levels were significantly increased in more than 20 types of tumor tissues. Elevated WDHD1 expression was associated with significantly shorter overall survival (OS) in 10 tumors. Furthermore, in uterine corpus endometrial carcinoma (UCEC) and liver hepatocellular carcinoma (LIHC), WDHD1 expression was significantly associated with higher histological grades and pathological stages. In addition, WDHD1 had a high diagnostic value among 16 tumors (area under the ROC curve [AUC] > 0.9). Functional enrichment analyses suggested that WDHD1 probably participated in many oncogenic pathways such as E2F and MYC targets (false discovery rate [FDR] < 0.05), and it was involved in the processes of DNA replication and DNA damage repair (*p*.adjust < 0.05). WDHD1 expression also correlated with the half-maximal inhibitory concentrations (IC50) of rapamycin (4 out of 10 cancers) and paclitaxel (10 out of 10 cancers). Overall, WDHD1 was negatively associated with immune cell infiltration and might promote tumor immune escape. Our analysis of genomic alterations suggested that WDHD1 was altered in 1.5% of pan-cancer cohorts and the “mutation” was the predominant type of alteration. Finally, through correlation analysis, we found that WDHD1 might be closely associated with tumor heterogeneity, tumor stemness, mismatch repair (MMR), and RNA methylation modification, which were all processes associated with the tumor progression.

**Conclusions:**

Our pan-cancer analysis of WDHD1 provides valuable insights into the genomic characterization and biological functions of WDHD1 in human cancers and offers some theoretical support for the future use of WDHD1-targeted therapies, immunotherapies, and chemotherapeutic combinations for the management of tumors.

**Supplementary Information:**

The online version contains supplementary material available at 10.1186/s12957-023-03187-3.

## Introduction

Worldwide, cancer continues to be the leading cause of premature mortality and a significant obstacle to increasing life expectancy [1, 2]. Current trends indicate a rise in cancer incidence and associated mortality, which can be attributed in part to population growth and aging [1]. It is projected that by 2070, there will be approximately 34 million new cancer cases diagnosed, twice the number recorded in 2018 [2]. While considerable progress has been made in understanding the causes of cancer over the past decade, there are still numerous unknowns concerning the mechanisms that initiate cancer development. Studies have turned their focus to investigating the mechanisms of tumorigenesis by examining the typical features of various human malignant tumors [3]. The concept of pan-cancer analysis is gaining prominence as it enables the identification of tumor biomarkers and signal pathways, leading to a better comprehension of tumorigenesis and progression at the molecular level [4, 5]. Therefore, the identification of biomarkers with diagnostic, prognostic, or therapeutic value from a pan-cancer perspective holds the potential to significantly enhance conventional cancer treatment and provide a formidable weapon against cancer [6].

WD repeat and high-mobility group box DNA binding protein 1 (WDHD1), also known as acidic nucleoplasmic DNA binding protein 1 (AND-1), and human chromosome transmission fidelity factor 4 (CTF4), is a relatively evolutionarily conserved protein with 1129 amino acids and homologs in the majority of eukaryotes [7]. The identification of WDHD1 initially occurred through the screening of mutations affecting chromosome transmission fidelity [8]. Subsequent studies have revealed that WDHD1 plays a crucial role in DNA replication, genomic stability, and DNA damage repair [9–11]. Considering tumorigenesis involves excessive DNA replication and genomic instability, it is imperative to comprehend the impact of WDHD1 on cancer development and progression. A recent study suggests that WDHD1 regulates the cancer cell cycle checkpoint, participates in oncogene-induced re-replication, and influences tumor growth and metastasis [12]. Furthermore, WDHD1 has been implicated as a ubiquitin ligase in tumorigenesis and malignant phenotypes [13, 14]. Despite the potential benefit of targeting WDHD1 for tumor diagnosis and treatment, only a few cancers have been associated with WDHD1, and to the best of our knowledge, its role in various types of cancer has not been explored.

Pan-cancer analysis has the potential to uncover common pathogenic mechanisms and signaling pathways shared among various types of cancer. This allows for the extension of effective treatments from one cancer to others that share similar genomic features, thereby identifying potential therapeutic targets that can be applied across various types of cancer [3, 15]. However, previous studies investigating the role of WDHD1 in cancer were limited to specific types of cancer, failing to reveal the shared genomic features, pathogenic mechanisms, and signaling pathways associated with WDHD1 across various types of cancer [16–18]. In contrast, our current study provides a comprehensive and panoramic view of WDHD1 across 33 different types of cancer. This includes genomic characterization, assessment of its clinical value, analysis of drug sensitivity, evaluation of the tumor immune microenvironment (TIME), and functional enrichment analysis. These findings offer a more comprehensive understanding of the potential role of WDHD1 in cancer and offer valuable insights for future clinical research and therapeutic approaches centered around WDHD1.

## Materials and methods

### Data processing

The RNA-sequencing data of a pan-cancer cohort (*n* = 15776) incorporated in this study was downloaded from the University of California Santa Cruz (UCSC) Xena website (https://xenabrowser.net/datapages/), which included different types of cancerous and normal tissues from TCGA (The Cancer Genome Atlas) and the Genotype-Tissue Expression (GTEx) [19]. The whole data was filtered to delete the missing and duplicate results and the transcripts per million (TPM) normalized expression spectrum data uniformly processed by the toil pipeline was log (TPM + 1) transformed for further analysis via using the rms function in the R package [20–23]. For the sake of clarity, we have divided all 33 cancer types into five categories, namely those of genito-urinary origin including bladder urothelial carcinoma (BLCA); kidney chromophobe (KICH); kidney renal clear cell carcinoma (KIRC); kidney renal papillary cell carcinoma (KIRP); breast invasive carcinoma (BRCA); endocervical adenocarcinoma (CESC); ovarian serous cystadenocarcinoma (OV); uterine corpus endometrial carcinoma (UCEC); uterine carcinosarcoma (UCS); testicular germ cell tumors (TGCT); prostate adenocarcinoma (PRAD); those of gastro-intestinal origin including cholangiocarcinoma (CHOL), colon carcinoma (COAD), esophageal carcinoma (ESCA), liver hepatocellular carcinoma (LIHC), pancreatic adenocarcinoma (PAAD), rectum adenocarcinoma (READ), and stomach adenocarcinoma (STAD); those of brain origin including glioblastoma multiforme (GBM) and brain lower grade glioma (LGG); those of head, neck, and lung origin including head and neck squamous cell carcinoma (HNSC), oral squamous cell carcinoma (OSCC), lung adenocarcinoma (LUAD), lung squamous cell carcinoma (LUSC); and other sites including adrenocortical carcinoma (ACC), diffuse large B cell lymphoma (DLBC), acute myeloid leukemia (LAML), mesothelioma (MESO), pheochromocytoma and paraganglioma (PCPG), sarcoma (SARC), skin cutaneous melanoma (SKCM), thyroid carcinoma (THCA), thymoma (THYM), and uveal melanoma (UVM).

We used the UALCAN (http://ualcan.path.uab.edu) to evaluate the WDHD1 protein expression in ten cancers, including BRCA, colon cancer, GBM, HNSC, KIRC, LIHC, LUAD, OV, PAAD, and UCEC using the “CPTAC analysis” module. In addition, the immunohistochemistry images of WDHD1 protein of 15 types of tumor tissues and their normal counterparts (detailed information in Fig. 2) were obtained from the atlas module of “pathology” and “tissue” of the Human Protein Atlas (HPA) database (https://www.proteinatlas.org), which maps the human proteome in cells, tissues, and organs with a variety of genomics techniques [24]. The antibody used for immunohistochemical staining was HPA001122.

This study used R (v3.6.3) software for statistical analysis. The Wilcoxon rank-sum test was used to determine the WDHD1 mRNA expression difference between unpaired normal and tumor tissues. The Wilcoxon signed-rank test detected the WDHD1 mRNA expression between paired tumors and normal tissues. The correlation between the two groups was determined by Spearman’s method. The *p* value for statistical significance was less than 0.05.

### The survival analysis of WDHD1 in pan-cancer

We filtered samples with less than 30 days of follow-up and without clinical information. Univariate Cox proportional hazards regression models were used in the hypothesis test that calculated the hazard ratios (HR, with 95% confidence interval [CI]) and *p* values for determining whether WDHD1 expression (median value) correlated with patient survival outcomes, including overall survival (OS), disease-specific survival (DSS), and progress-free interval (PFI) in each tumor [25]. The R package “forest” displayed the results of the prognostic analysis. The Kaplan–Meier (K-M) plots evaluated patient survival status in specific cancers with the R packages “survival” (for statistical analysis) and “survminer” (for visualization) [26, 27]. In addition, we assessed the association between WDHD1 expression and patient prognosis using the PrognoScan database (http://www.prognoscan.org/) and independent datasets from the Gene Expression Omnibus (GEO) database (https://www.ncbi.nlm.nih.gov/geo/) [28, 29]. A *p* value of less than 0.05 was considered statistically significant.

### Receiver operator characteristic (ROC) curve of WDHD1 in pan-cancer

Based on ROC curves, the predictive accuracy (defined here as diagnostic value) of using WDHD1 expression to discriminate between tumor tissues and normal tissues in pan-cancer was estimated. The WDHD1 mRNA expression in the tumor and corresponding normal tissues from GTEx and TCGA was used to construct ROC curves based on sensitivity and specificity. The R packages “pROC” and “ggplot2” were used to calculate and plot the ROC curves [30]. There was a range of 0.5 to 1 AUC (area under the ROC curve). As the AUC approaches 1, its diagnostic value increases. In the range of 0.5–0.7, AUC had low accuracy. The accuracy of AUC was certain between 0.7–0.9 and high between 0.9 and 1.0.

### Protein–protein interaction analysis and functional enrichment analysis of WDHD1

GeneMANIA offered us an approach to acquiring 20 genes that may interact with WDHD1. We input “WDHD1” and built a functional protein–protein interaction network. We also obtained the top 200 genes co-expressed with WDHD1 in all cancers by gene expression profiling interactive analysis 2 (GEPIA2). After taking the intersection of WDHD1-interacted genes and WDHD1-coexpressed genes using the Venn diagram, we identified CDC25A and POLE2. The correlation of WDHD1 with co-expressed genes in pan-cancer was visualized by TIMER2.0. Functional enrichment analysis, including BP (biological process), CC (cellular component), MF (molecular function), and KEGG (Kyoto Encyclopedia of Genes and Genomes) using interacted and co-expressed genes with the help of the R package “ClusterProfiler” [31].

Using gene set enrichment analysis (GSEA), we investigated different biological and oncogenic signaling pathways between WDHD1-high and low groups based on median WDHD1 value from the TCGA cohorts. “ClusterProfiler” was used to carry out enrichment analyses on the MSigDB H (hallmark gene set) and C6 (oncogenic signature gene set) gene sets. The enrichment significance of gene sets was determined by *p*.adjust < 0.05, FDR < 0.25, and |NES|> 1.

### Chemotherapy drug sensitivity analysis

In order to assess each sample’s response to rapamycin and paclitaxel, we used the publicly accessible pharmacogenomics database The Genomics of Drug Sensitivity in Cancer (GDSC). Ridge regression was used to estimate the samples’ half-maximal inhibitory concentrations (IC50). Based on Wilcoxon rank sum tests, we compared the IC50 between groups of WDHD1 high and low. We considered the result statistically significant if the *p* < 0.05.

### Analysis between WDHD1 and tumor immunity

An analysis of the relationship between WDHD1 expression and immune cell infiltration was conducted using two algorithms named single sample GSEA (ssGSEA) and Estimate. The former algorithm used specific markers of each kind of immune cell as gene sets to calculate the enrichment score of each sample, thus inferring the infiltration of immune cells in each sample [32]. The latter provided built-in markers to calculate the immune, stromal, and estimate scores. R packages “ggplot2” and “ggpubr” helped us to deal with this process.

Moreover, we examined the co-expression of WDHD1 and immune-related genes, including immunostimulators, immunoinhibitors, histocompatibility complex (MHC) molecules, chemokines, and chemokine receptors across different cancers. Statistical significance was determined by *p* values < 0.05 in Spearman’s correlation. We visualized the correlations as heatmaps using the “ggplot2” package.

### The genetic alteration analysis of WDHD1 in pan-cancer

The genetic alteration information of WDHD1 was explored through cBioPortal (https://www.cbioportal.org/) [33]. We incorporated all TCGA Pan-Cancer Atlas studies into our study. The “Cancer Type Summary,” “Mutations,” and “mRNA vs Study” modules were utilized to get the genetic alteration information of WDHD1 in the pan-cancer cohort. The “Survival” module was used to get the survival difference of patients with or without WDHD1 mutation.

### Tumor heterogeneity and stemness analysis of WDHD1

We downloaded the simple nucleotide variation dataset of level 4 for all TCGA samples processed by MuTect2 software [34]. We calculated the tumor mutation burden (TMB) scores of each sample using the R package “Maftools” (version 2.8.05). The microsatellite instability (MSI), homologous recombination deficiency (HRD), and loss of heterozygosity (LOH) scores of each tumor patient were acquired from previous research [35, 36]. In stemness analysis, the mRNA and DNA methylation profiles were used to calculate four stemness scores, including DNA methylation-based stemness score (DNAss), RNA expression-based stemness score (RNAss), epigenetically regulated DNA methylation-based stemness score (EREG-METHss), and epigenetically regulated RNA expression-based stemness score (EREG.EXPss) on the base of a previous study [37]. Spearman’s method determined the correlations between WDHD1 expression with tumor heterogeneity and tumor stemness.

### CancerSEA

We investigated the functional states of WDHD1 with the help of CancerSEA, a publicly available database that provides access to decoding cancer cell functions at the single-cell level [38]. An investigation was conducted into the correlations between WDHD1 and 14 different functional states (angiogenesis, apoptosis, cell cycle, differentiation, DNA damage, DNA repair, epithelial-mesenchymal transition [EMT], hypoxia, inflammation, invasion, metastasis, proliferation, quiescence, and stemness) in 15 cancer types. For the correlation coefficient, a threshold of 0.3 was used, and for the significant difference, a threshold of 0.05 was used.

## Results

### Differential expression of WDHD1 across various types of cancer

**Fig. 1:**
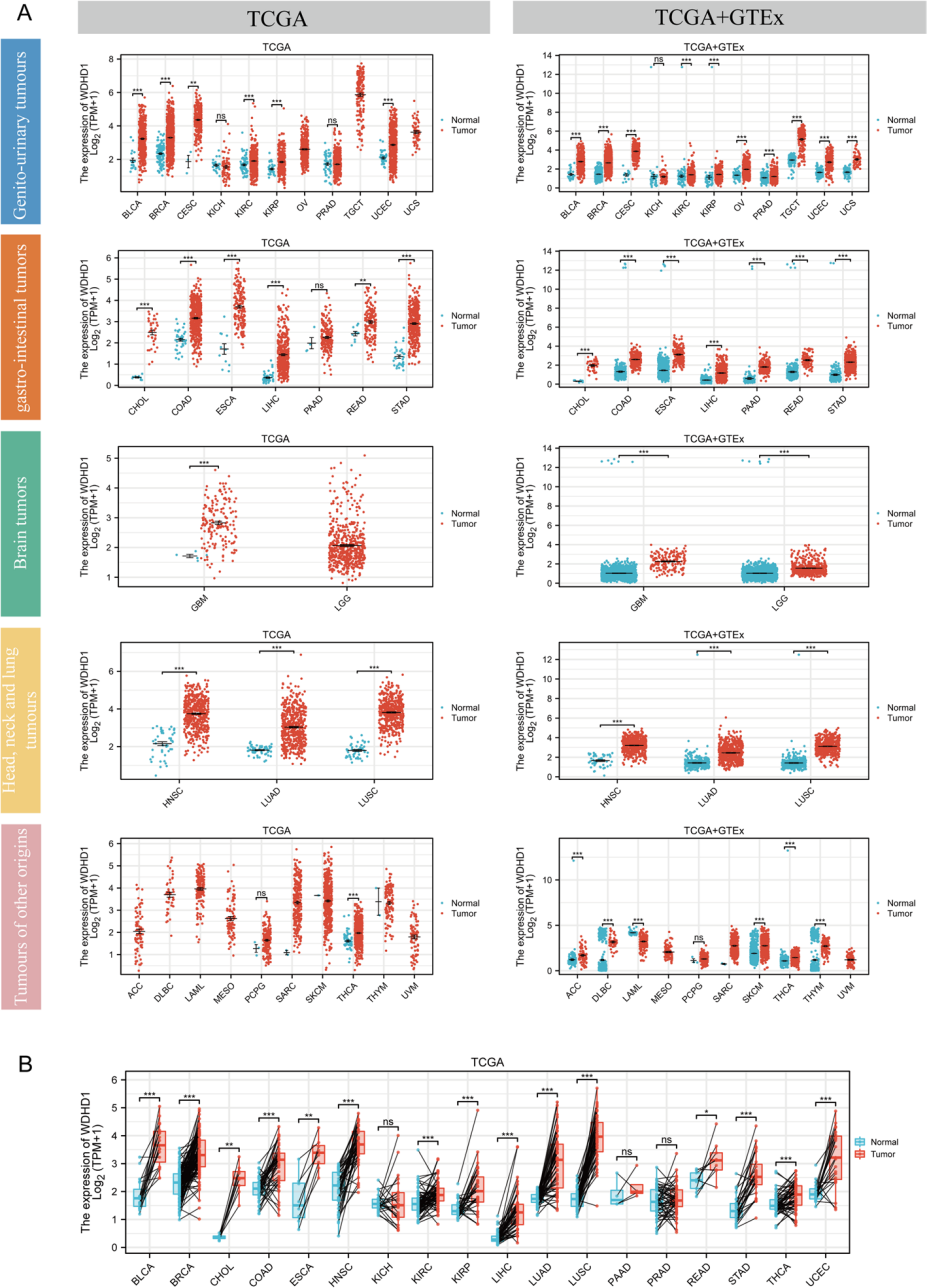
WDHD1 mRNA expression is generally elevated in tumor tissues of different origins compared to corresponding normal tissues. **A** WDHD1 mRNA expression difference between tumor and normal tissues in the TCGA (left). The expression of WDHD1 mRNA differs between tumor tissues from the TGCA and normal tissues from the TGCA and GTEx (right). Tumors have been categorized according to their origins and are indicated by bars with different colors (The subsequent figures in the article are also labeled as such for categorization). **B** WDHD1 mRNA expression in TCGA tumor and paired adjacent normal tissues (*p < 0.05, **p < 0.01, ***p < 0.001, ns, not statistically significant)

Firstly, we examined WDHD1 mRNA expression across various types of cancer. Using expression data from cancerous and paracancerous tissues sourced from the TCGA, we observed a significant elevation of WDHD1 mRNA expression levels in cancer tissues across various origins when compared to normal tissues (Fig. 1A, left panel). However, due to a lack of sufficient paracancerous data, analysis for OV, TGCT, UCS, LGG, ACC, DLBC, LAML, MESO, SARC, SKCM, THYM, and UVM could not be conducted. No expression differences were observed in KICH, PRAD, PAAD, and PCPG. Comparing patients with genito-urinary tumors to normal samples from both the TCGA and GTEx databases, significantly higher WDHD1 expression was observed, with the exception of KICH. For gastrointestinal, brain, head, neck, and lung tumors, WDHD1 expression was significantly elevated across all types of cancers. Similarly, increased WDHD1 expression was found in ACC, DLBC, SKCM, THCA, and THYM tumors of other origins. However, lower WDHD1 mRNA expression was observed in LAML (Fig. 1A, right panel). Among the paired samples from 18 cancers, WDHD1 mRNA expression was increased in BLCA, BRCA, CHOL, COAD, ESCA, HSNC, KIRC, KIRP, LIHC, LUAD, LUSC, RAED, STAD, THCA, and UCEC (Fig. 1B). Additionally, we validated WDHD1 mRNA expression using large GEO datasets across 20 cancers (Figures S1 and S2). The results consistently showed ubiquitous upregulation of WDHD1 in cancerous tissues.

**Fig. 2:**
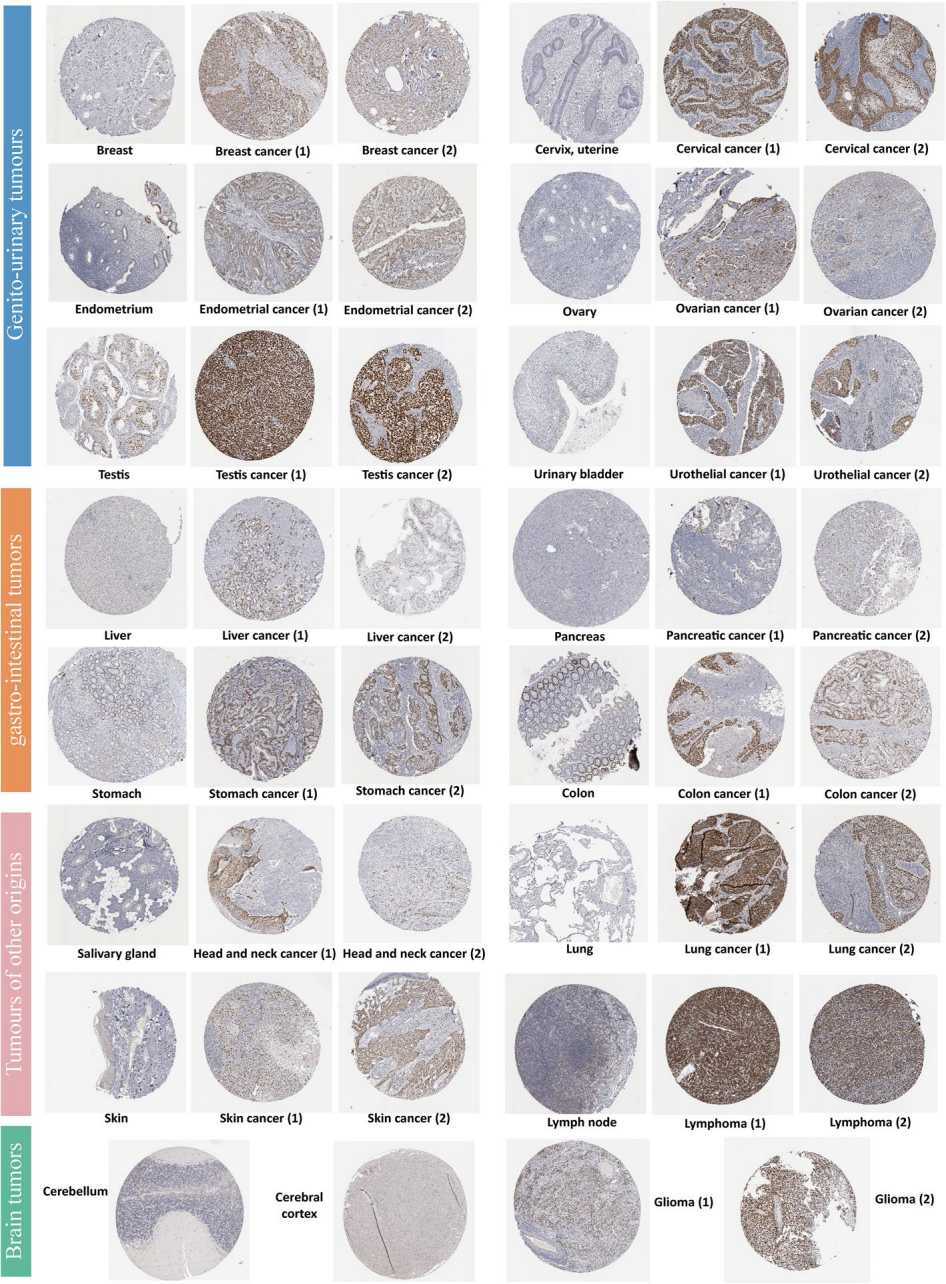
Normal (left) and tumor (right) immunohistochemical images show differential WDHD1 protein expression

Biological functions are widely recognized to be mediated through the protein expression of genes. Therefore, we extended our investigation beyond mRNA levels and explored WDHD1 protein expression in 10 different types of cancer. The analysis revealed a significant upregulation of WDHD1 protein in BRCA, COAD, glioblastoma multiforme (GBM), HNSC, KIRC, LIHC, LUAD, PAAD, and UCEC when compared to normal tissues. In OV, although WDHD1 protein expression was elevated, the result was not statistically significant (Figure S3). Additionally, we used the HPA database to obtain immunohistochemical images of WDHD1 protein expression in normal and tumor tissues. These images clearly demonstrated a significant increase in WDHD1 protein expression in 15 cancers when compared to normal tissues (Fig. 2). In summary, both the WDHD1 mRNA and protein expression levels exhibited a general increase across various types of tumors of different origins.

### The prognostic and diagnostic value of WDHD1 across various types of cancer

**Fig. 3:**
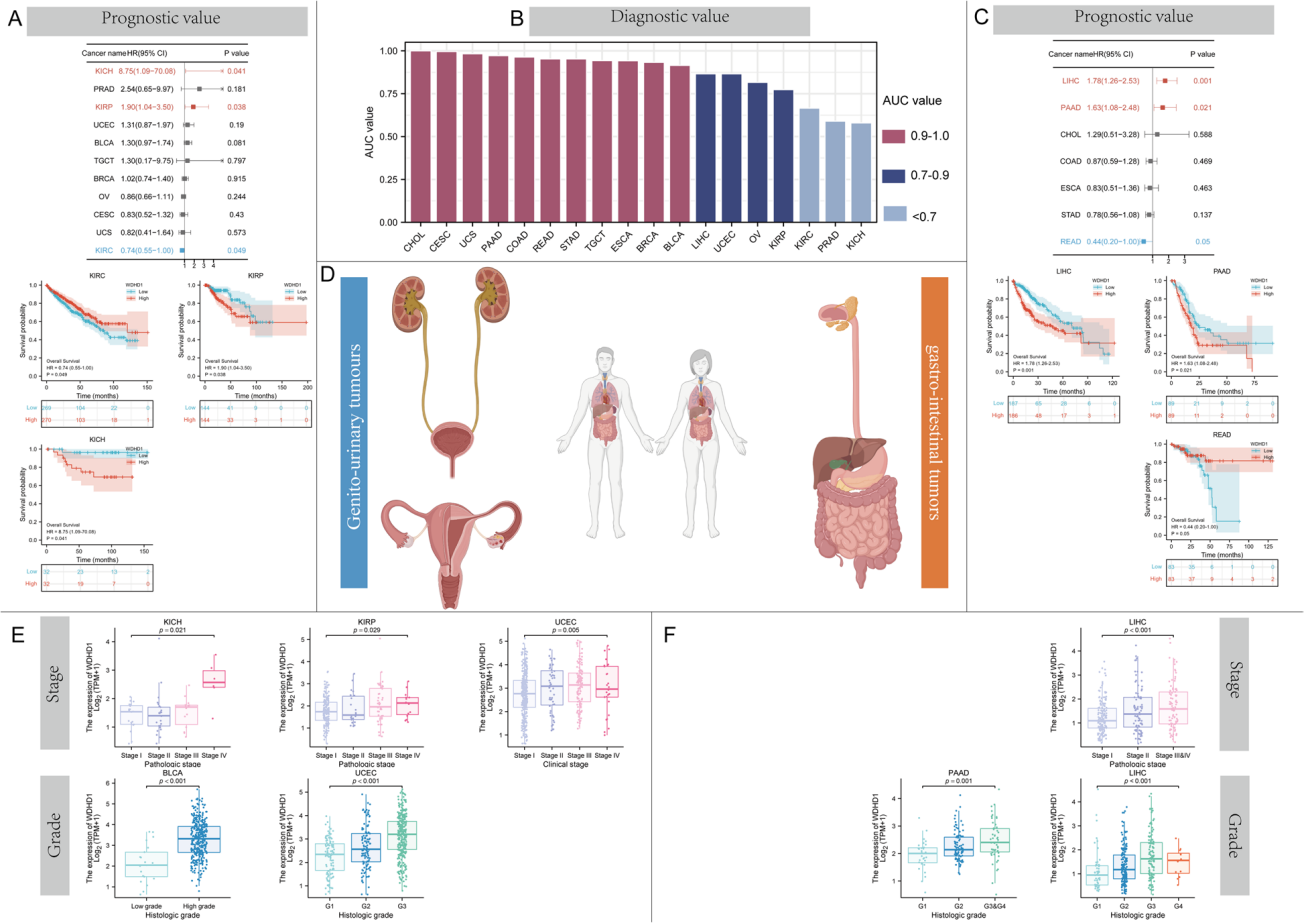
The clinical value of WDHD1 in gastro-intestinal and genito-urinary tumors. The prognostic value of WDHD1 in gastro-intestinal (**A**) and genito-urinary (**C**) tumors. **B** The diagnostic value of WDHD1 in these tumors. **D** A brief illustration of tumors of two origins. WDHD1 expression correlates with the grading and staging of tumors of genito-urinary (**E**) and gastro-intestinal (**F**) origins

We conducted an analysis of the clinical significance of WDHD1 in relation to the origins of the tumors. To investigate the association between WDHD1 expression and patient prognosis, we made proportional hazard assumptions (the detailed information was shown in Table S1, all *p* > 0.05) prior to conducting the Cox regression analysis, and then generated K-M survival curves for OS, DSS, and PFI. In the case of genito-urinary tumors (Fig. 3D), high WDHD1 expression was associated with improved OS in KIRC (*p* = 0.049), while it was associated with worse OS in KIRP (*p* = 0.038) and KICH (*p* = 0.041) (Fig. 3A). Moreover, WDHD1 was found to be associated with a higher pathological stage in KICH, KIRP, and UCEC, as well as a higher histological grade in BLCA and UCEC (Fig. 3E). For gastrointestinal tumors (Fig. 3D), we found that WDHD1 played a detrimental role in LIHC (*p* = 0.001) and PAAD (*p* = 0.021), while it exhibited a protective role in READ (*p* = 0.050) (Fig. 3C). Similarly, WDHD1 expression showed an increase with the advancing stage of LIHC. Additionally, higher WDHD1 expression was observed in patients with higher tumor grades in LIHC and PAAD (Fig. 3F). The diagnostic accuracy of WDHD1 in these tumors was assessed using the ROC curves. Among the 18 types of tumors, 15 showed ROC curves with AUC values exceeding 0.7, and 11 of them surpassed 0.9, indicating the robust diagnostic value of WDHD1 in these tumors (Fig. 3B).

**Fig. 4:**
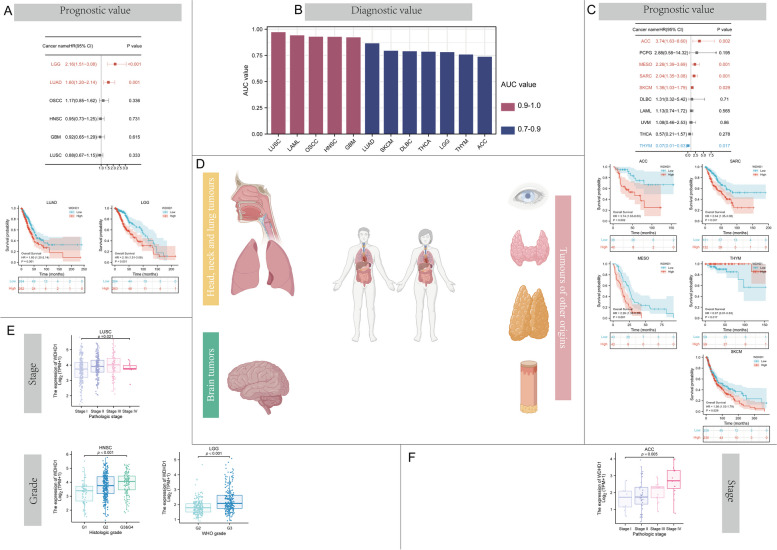
The clinical value of WDHD1 in gastro-intestinal and genito-urinary tumors. The prognostic value of WDHD1 in gastro-intestinal (**A**) and genito-urinary (**C**) tumors. **B** The diagnostic value of WDHD1 in these tumors. **D** A brief illustration of tumors of two origins. WDHD1 expression correlates with the grading and staging of tumors of genito-urinary (**E**) and gastro-intestinal (**F**) origins

Regarding head, neck, lung, and brain tumors (Fig. 4D), high WDHD1 expression was associated with shorter OS in LUAD (*p* = 0.001) and LGG (*p* < 0.001) (Fig. 4A). Additionally, the analysis revealed that WDHD1 expression was associated with a higher stage in LUSC and a higher grade in HNSC and LGG (Fig. 4E). In tumors of other origins, high WDHD1 expression indicated an unfavorable prognosis for patients with ACC (*p* = 0.002), MESO (*p* = 0.001), SARC (*p* = 0.001), and SKCM (*p* = 0.029), and a favorable prognosis for patients with THYM (*p* = 0.017) (Fig. 4C). Furthermore, more advanced tumor stages were associated with higher WDHD1 expression in ACC (Fig. 4F). The diagnostic value of WDHD1 was evaluated in the tumors indicated in Fig. 4B. Among the 12 types of cancer, all the ROC curves had AUC values exceeding 0.7, indicating that WDHD1 expression demonstrated moderate to strong efficacy in distinguishing tumors from normal tissue (Fig. 4B). To provide a comprehensive overview of the diagnostic value of WDHD1, all ROC curves for the 30 types of cancer were shown in Figure S4. Additionally, Table S2 provided more exhaustive information of the ROC curves, including positive predictive value, negative predictive value, and the Youden index.

As an external validation, the diagnostic value of WDHD1 was analyzed in detail in 41 independent GEO datasets (over 20 cancer types). Among them, only 3 datasets had AUC values of ROC curves lower than 0.7 (Figure S5), which was highly consistent with the analysis results of the TCGA database, indicating that WDHD1 has a certain to high diagnostic value in a wide range of tumor types. Details of the ROC curves for these GEO external validation datasets were shown in Table S2.

The association of WDHD1 with DSS (Figure S6) and PFI (Figure S7) in patients with tumors was also examined. Notably, WDHD1 expression was found to significantly predict all survival indicators (OS, DSS, and PFI) in seven types of tumors, including those with the gastrointestinal tract (LIHC and PAAD), lung (LUAD), brain (LGG), and other origins (ACC, MESO, and SARC). This suggests that WDHD1 holds good prognostic value for these specific types of cancer.

To validate the survival results obtained from the TCGA, we used datasets from the GEO and PrognoScan as a complementary approach. Figure S8 was composed of the K-M survival results derived from 26 independent GEO datasets. Additionally, we incorporated data from 27 different cohorts from PrognoScan (Figures S9 and 10). The survival analysis shown in Figures S8–S10 consistently indicated that high WDHD1 expression was strongly associated with poorer survival status among patients with ACC, BLCA, BRCA, brain cancer, blood cancer, lung cancer, PAAD, SARC, and SKCM. These findings align with the prognostic results from the TCGA dataset, thus highlighting the potential diagnostic and prognostic value of WDHD1 across various types of cancer. Moreover, the correlation observed between WDHD1 and the grading and staging of tumors from various origins suggests a potential involvement of WDHD1 in the progression of these tumors.

### Functional enrichment and drug sensitivity analyses of WDHD1 across various types of cancer

**Fig. 5:**
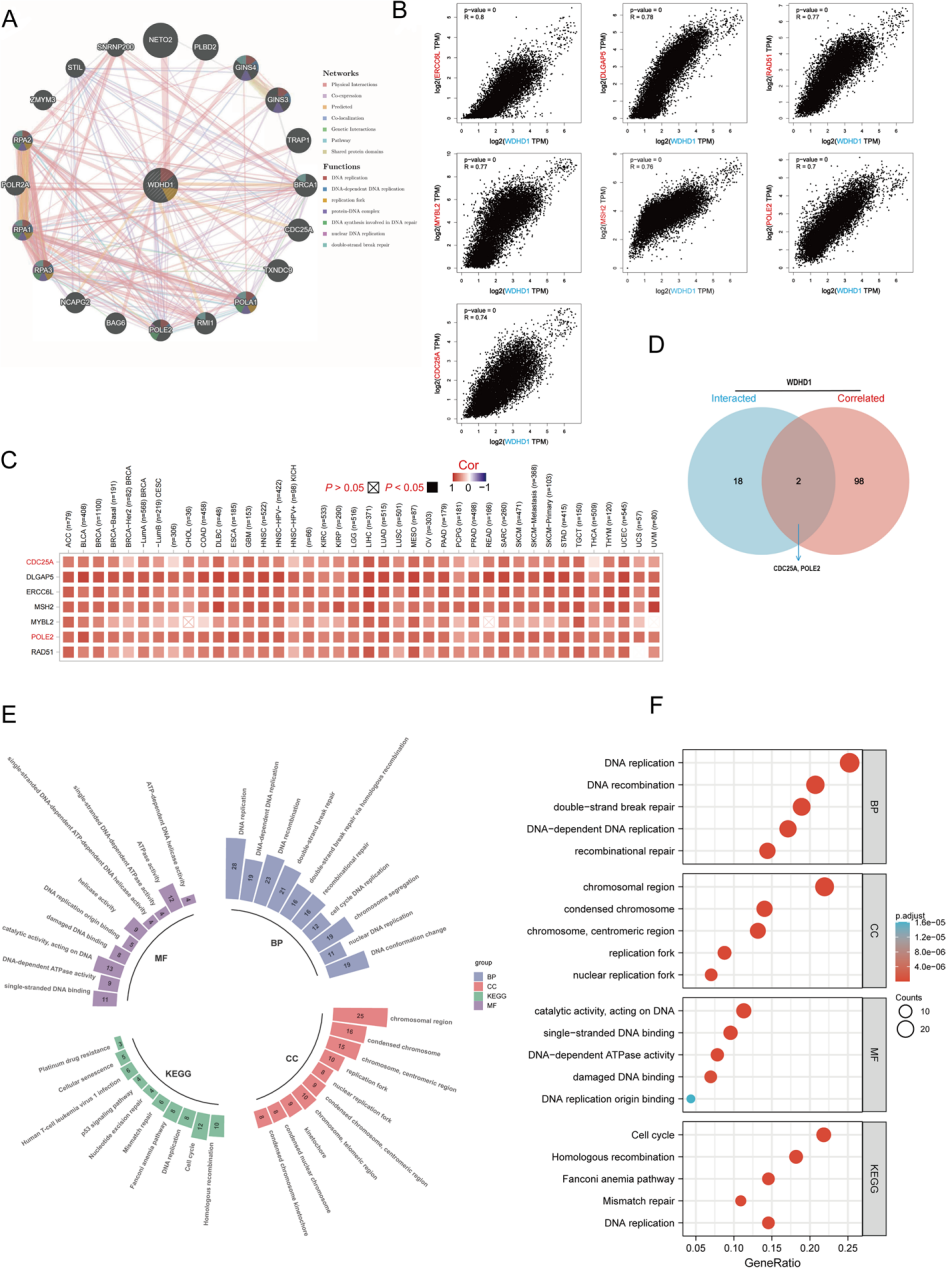
Functional enrichment analysis of WDHD1 co-expressed and interacted genes. **A** This network shows the protein–protein interactions between WDHD1 and co-expressed genes. Networks are color-based on their functions. The correlation between WDHD1 and ERCC6L, DLGAP5, RAD51, MYBL2, MSH2, POLE2, and CDC25A in pan-cancer is shown in the scatterplots (**B**) and a heatmap (**C**). **D** VENN plot showing the intersection of WDHD1 co-expressed and interacted genes. Functional enrichment analysis is presented as a circular barplot (**E**) and bubble plot (**F**)

To further explore the role of WDHD1 in tumor progression, we conducted an enrichment analysis using genes that co-express or interact with WDHD1. Initially, we constructed the protein–protein interaction (PPI) network using 20 genes known to interact with WDHD1. Figure 5A shows that these WDHD1-interacting genes are actively involved in crucial processes such as DNA replication, replication forks, protein-DNA complexes, and DNA repair. Subsequently, utilizing GEPIA2, we obtained the top 100 genes co-expressed with WDHD1. Among them, the five genes with the highest correlation coefficients were *ERCC6L* (*R* = 0.8, *p* < 0.0001), *DLGAP5* (*R* = 0.78, *p* < 0.0001), *RAD51* (*R* = 0.77, *p* < 0.0001), *MYBL2* (*R* = 0.77, *p* < 0.0001), and *MSH2* (*R* = 0.76, *p* < 0.0001) (Fig. 5B). By intersecting WDHD1-interacted and WDHD1-correlated gene sets, we identified two genes named *CDC25A* and *POLE* (Fig. 5D). The Spearman’s correlations of these genes with WDHD1 were 0.74 and 0.70, respectively (Fig. 5B). Additionally, Fig. 5C presents a heatmap illustrating the correlation between WDHD1 and these seven genes (*ERCC6L*, *DLGAP5*, *RAD51*, *MYBL2*, *MSH2*, *CDC25A*, and *POLE*) within the TCGA pan-cancer cohort.

To gain insights into the biological function of WDHD1, we performed functional enrichment analysis using a combination of WDHD1-correlated and WDHD1-interacted genes. The results, depicted in Figs. 5E and F, indicate that the KEGG pathway analysis highlights the potential involvement of WDHD1 in “cell cycle,” “homologous recombination,” “mismatch repair (MMR),” and “DNA replication” processes, which are associated with its pro-carcinogenic roles. Furthermore, GO enrichment analysis revealed that these genes might be associated with the biological processes of “DNA replication,” “DNA recombination,” and “double-strand break repair.” Additionally, they may contribute to the CC of “chromosomal region,” “condensed chromosome,” and “chromosome, centromeric region.” Moreover, these genes were associated with MF such as “catalytic activity,” “single-stranded adenosine triphosphatase (ATPase) activity,” and “DNA-dependent ATPase activity.”

**Fig. 6:**
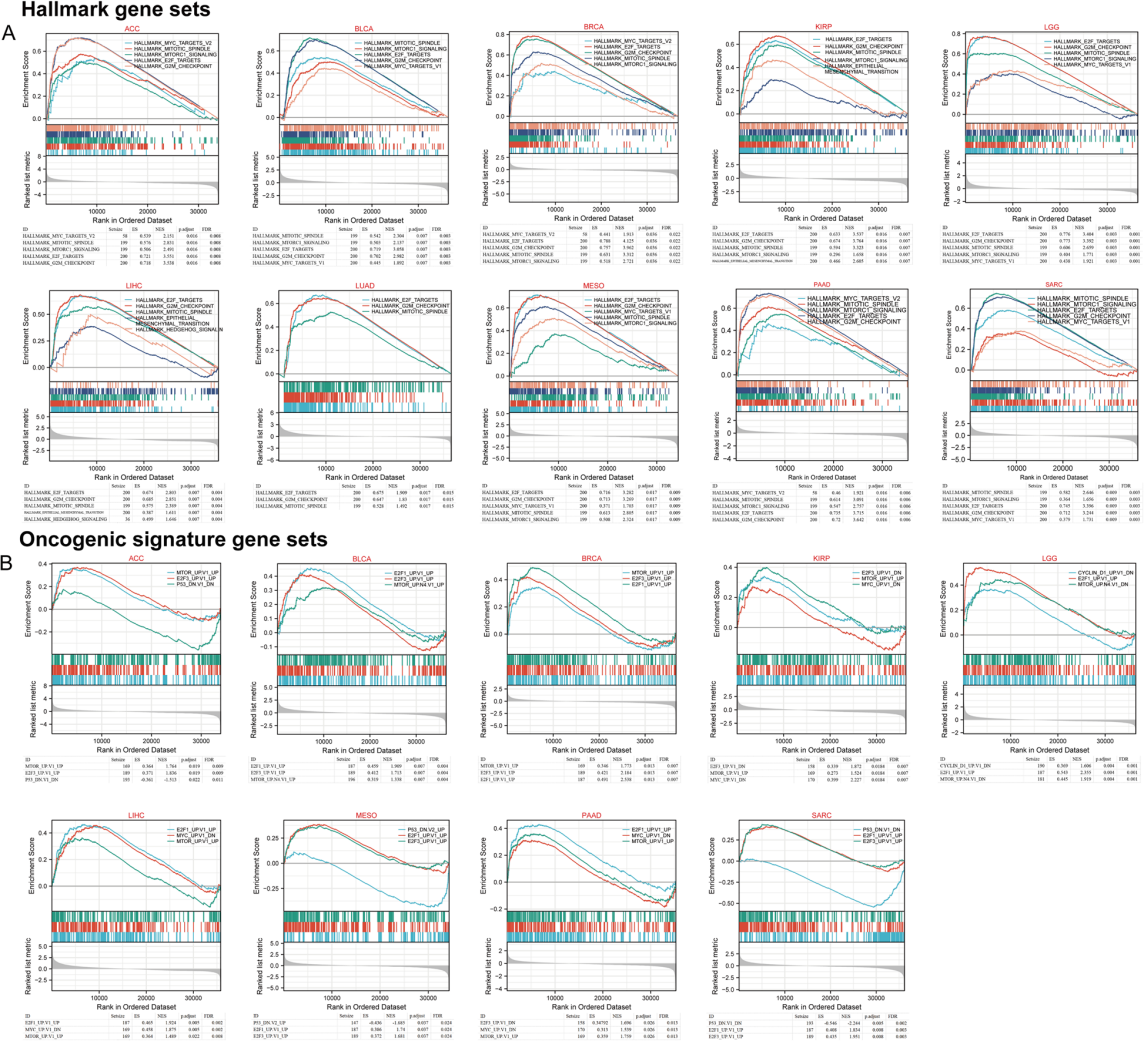
Gene set enrichment analysis of WDHD1. **A** Hallmark gene sets enriched in the WDHD1 high expression group. **B** Oncogenic signature gene sets enriched in the WDHD1 high expression group. ES is the enrichment score. NES is the normalized enrichment score, which considers the number and size of gene sets. FDR represents the false discovery rate

We aimed to gain a deeper understanding of the impact of WDHD1 on tumorigenesis. To achieve this, we performed GSEA in cancers where WDHD1 serves as a prognostic factor. By utilizing MSigDB H (hallmark gene sets), we conducted GSEA and observed a positive and significant correlation between high WDHD1 expression and genes involved in crucial processes such as assembly of the mitotic spindle, G2/M checkpoint, cell cycle-related E2F transcription targets, MYC targets, and the mammalian target of rapamycin complex 1 (mTORC1) signaling (Fig. 6A). Furthermore, the enrichment analysis based on C6 (oncogenic gene sets) revealed that high WDHD1 expression was associated with numerous signature oncogenes, including *E2F*, *MYC*, and *mTOR*. Contrastingly, low WDHD1 expression was associated with the tumor suppressive gene *p53* (Fig. 6B).

**Fig. 7:**
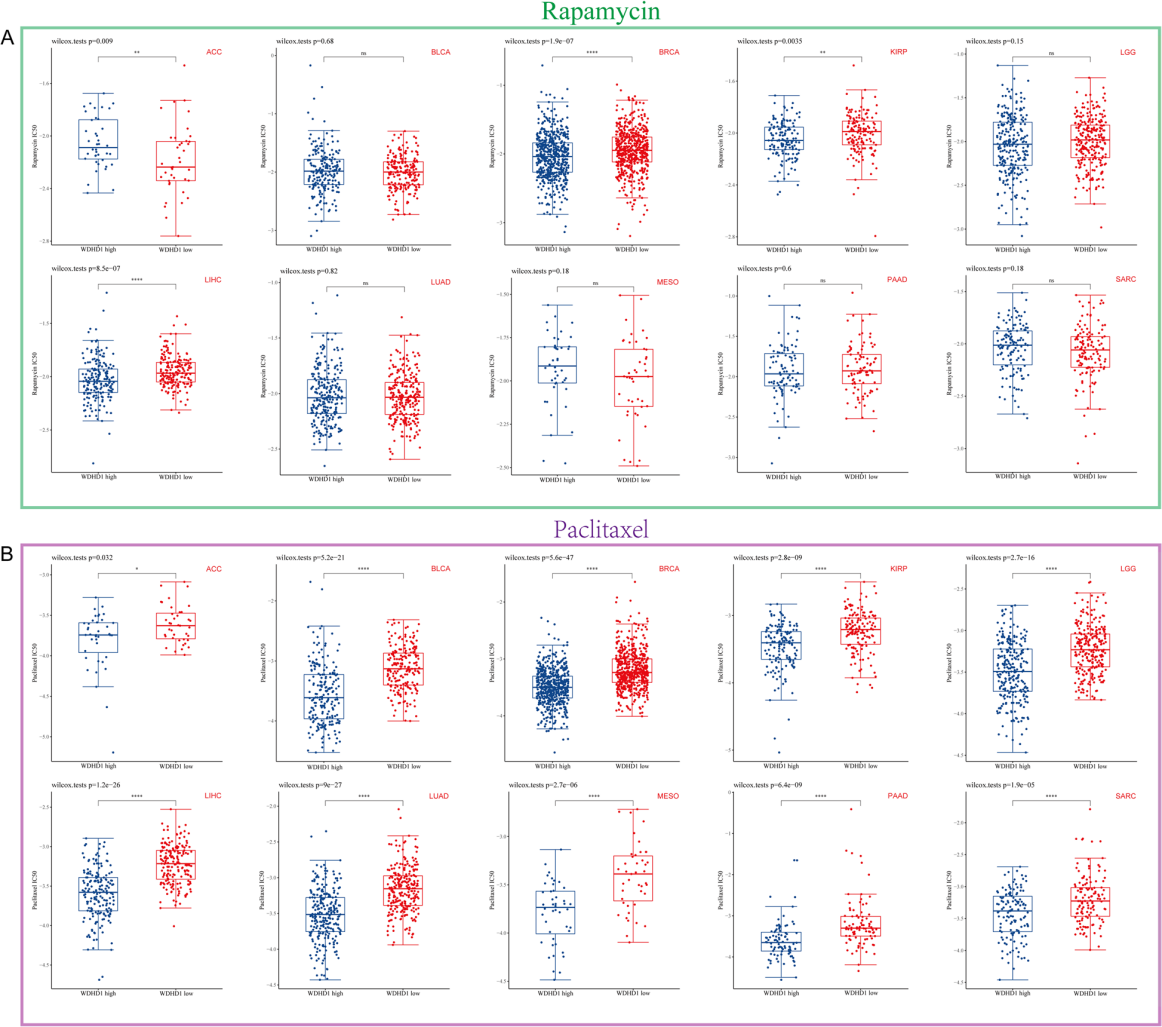
Drug sensitivity analysis of WDHD1. The association between WDHD1 expression and drug sensitivity of rapamycin (**A**) and paclitaxel (**B**) in ten cancers. The horizontal coordinate indicates the high and low WDHD1 expression groups, and the vertical coordinate indicates the distribution of the IC50 score. *p < 0.05, **p < 0.01, ****p < 0.0001, ns, not statistically significant

As previously mentioned, there is a significant association between WDHD1 and the mTOR signaling pathway as well as the G2/M checkpoint. Considering this, we selected two chemotherapy drugs, rapamycin and paclitaxel, for further investigation. Rapamycin, known for its ability to inhibit mTOR, has demonstrated efficacy in cancer treatment. On the other hand, paclitaxel exerts its therapeutic effects by disrupting the formation of a normal mitotic apparatus during the G2/M phase of the cell cycle, making it a valuable agent in cancer chemotherapy [39]. To establish a stronger clinical relevance of WDHD1, we evaluated its relationship with the IC_50_ values of rapamycin and paclitaxel. As shown in Fig. 7A, in BRCA, KIRP, and LIHC, patients with high WDHD1 expression demonstrated greater sensitivity to rapamycin treatment. Conversely, in ACC, patients with low WDHD1 expression responded more effectively to rapamycin treatment. In paclitaxel analysis, we found that the IC_50_ values of paclitaxel were significantly lower in the groups with high WDHD1 expression compared to those with low expression across all 10 types of cancer, suggesting that paclitaxel treatment may confer greater benefits to patients with high WDHD1 expression (Fig. 7B).

### The immunological roles of WDHD1 across various types of cancer

**Fig. 8:**
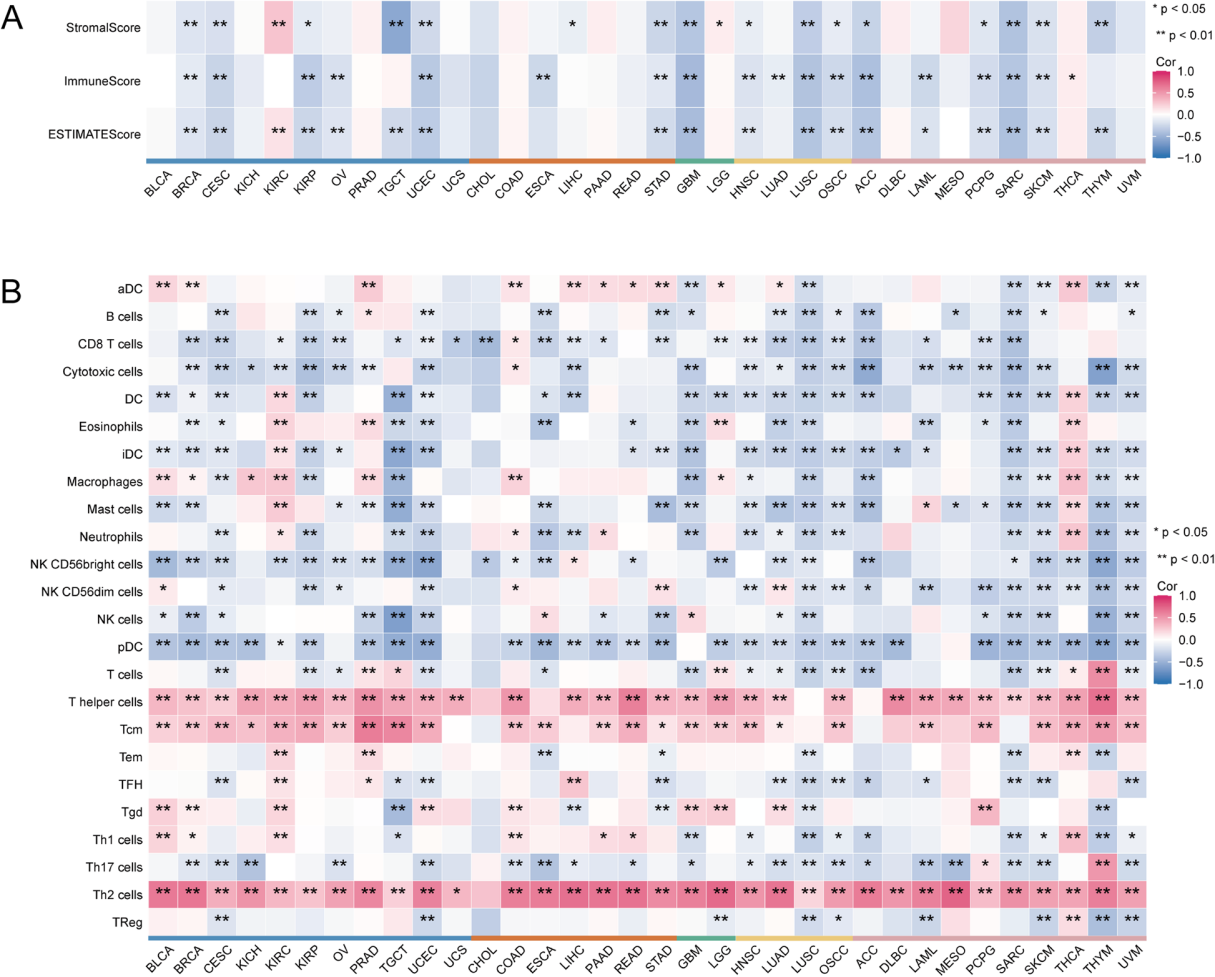
The association between WDHD1 expression and immune cell infiltration. The ESTIMATE algorithm (**A**) and ssGSEA algorithm (**B**) are used to determine the correlation between WDHD1 expression and immune infiltration (*p < 0.05, **p < 0.01). Tumors of different origins are indicated by bars with different colors

To investigate the relationship between WDHD1 and immune infiltration and regulation, we examined Spearman’s correlations between WDHD1 expression and immune cell infiltration levels as well as immunological biomarkers. Using the “estimate” package, we observed a consistent negative correlation between WDHD1 and stromal and immune infiltration across most cancers. Notably, in genito-urinary cancer (BRCA, CESC, TCGT, and UCEC); gastrointestinal tumor (STAD); brain tumor (LGG), head, neck, and lung tumors (HNSC, LUSC, and OSCC); and tumors of other origins (ACC, PCPG, SARC, and SKCM), WDHD1 expression showed significant negative correlations with StromalScore, ImmuneScore, and EstimateScore (Fig. 8A). Furthermore, the heatmap shown in Fig. 8B depicts significant and positive correlations between WDHD1 expression and the infiltration of T helper cells, central memory T cells (Tcm), and Th2 cells. Conversely, the infiltration of several other immune cells was negatively correlated with WDHD1 expression (Fig. 8B) in the pan-cancer analysis.

**Fig. 9:**
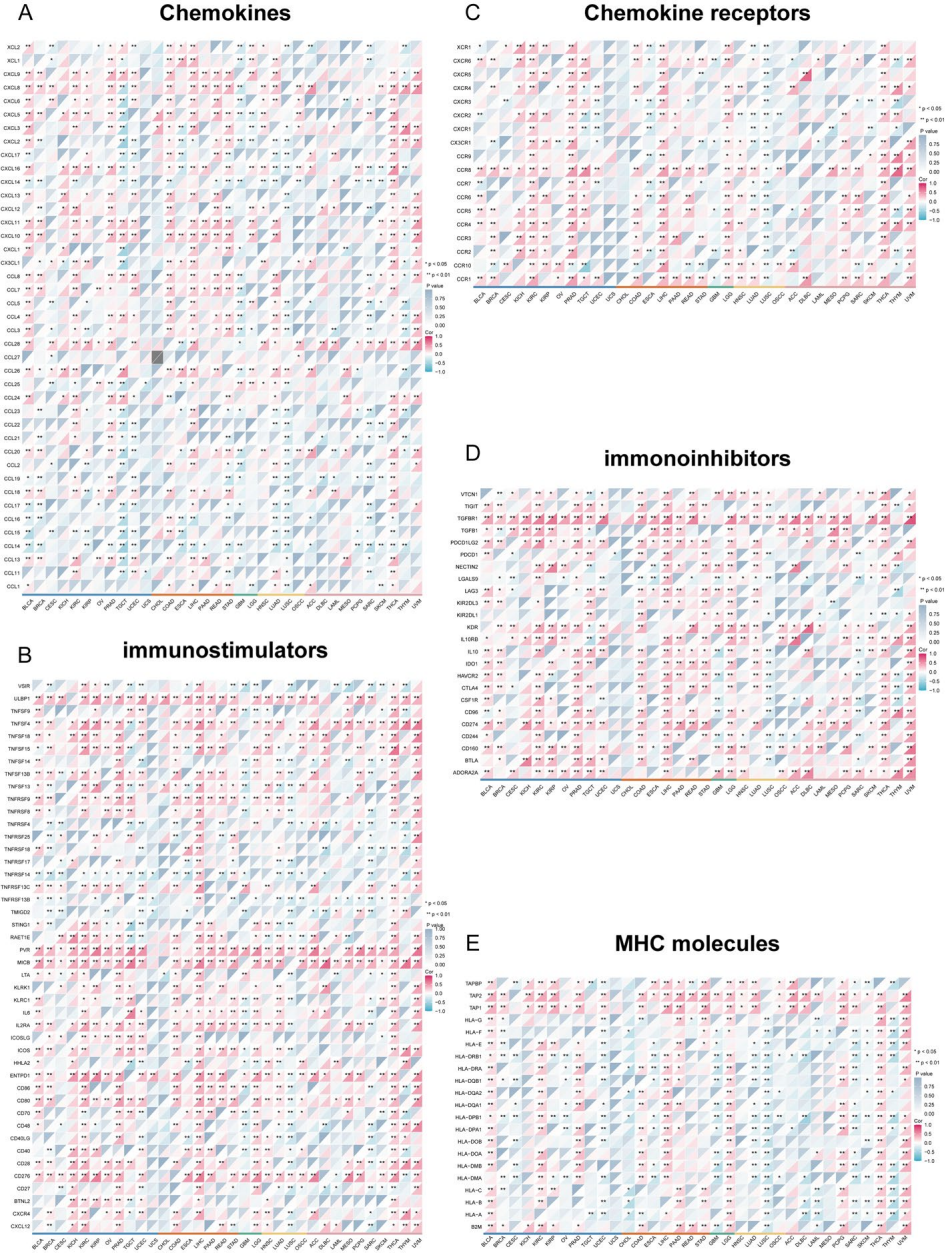
Correlation of WDHD1 with immunoregulatory genes in pan-cancer. Correlation between WDHD1 and chemokines (**A**), immunostimulators (**B**), chemokine receptors (**C**), immunoinhibitors (**D**), and MHC molecules (**E**). *p < 0.05, **p < 0.01

In our further investigation, we explored the association between WDHD1 and immunoregulatory genes. Across various types of cancer, positive correlations between WDHD1 expression and most chemokines were observed in BLCA, KIRC, COAD, LIHC, and THCA. Conversely, negative correlations between chemokines and WDHD1 expression were prevalent in TGCT, GBM, LUSC, and SARC (Fig. 9A). Generally, immunostimulators in human cancers exhibited a positive correlation with WDHD1, particularly in BLCA, KIRC, PRAD, COAD, LIHC, LGG, THCA, and UVM. Notably, two specific immunostimulators, PVR and MICB, demonstrated significant and positive correlations with WDHD1 across multiple types of cancer (Fig. 9B). Regarding chemokine receptors, positive correlations with WDHD1 were observed in most cases in KIRC, PRAD, LIHC, LGG, and THCA, whereas in LUSC, all correlations were negative (Fig. 9C). Intriguingly, WDHD1 also showed positive correlations with a majority of immunoinhibitors, particularly BLCA, KIRC, PRAD, COAD, LIHC, LGG, LUAD, and UVM (Fig. 9D). Furthermore, WDHD1 showed positive correlations with most major MHC molecules in BLCA, KIRC, LIHC, LGG, THCA, and UVM, while negative correlations were observed in UCEC, LUSC, SARC, and THYM (Fig. 9E). Collectively, these findings suggest that WDHD1 has significant correlations with various immunoregulatory genes in pan-cancer analysis, particularly in BLCA, KIRC, LIHC, LGG, and THCA, suggesting its potential active involvement in the immunomodulatory process within the TIME.

To gain better and deeper insights into the impact of WDHD1 on the response to immune checkpoint blockade (ICB) treatment, we evaluated the tumor immune dysfunction and exclusion (TIDE) scores of WDHD1 in five specific types of cancer: BLCA, LGG, LIHC, LUAD, and PAAD. These cancers have previously shown a positive correlation between WDHD1 expression and most immunoinhibitors. It has been well documented that patients with high TIDE scores tend to exhibit poor responses to ICB therapy and have shorter survival rates [40]. Notably, we observed a significant increase in TIDE scores within the high WDHD1 expression group in LGG (*p* < 0.0001, Figure S11B), LIHC (*p* < 0.0001, Figure S11C), LUAD (*p* < 0.0001, Figure S11D), and PAAD (*p* = 0.027, Figure S11E). In BLCA, although the result was marginally significant, patients with high WDHD1 expression also exhibited higher TIDE scores (*p* = 0.078, Figure S11A). Based on these findings, we hypothesize that WDHD1 may contribute to the suppression of the immune response and the promotion of immune escape. Targeting WDHD1 could potentially improve the outcomes of immunotherapy for these particular types of tumors. Additionally, we previously demonstrated that patients with high WDHD1 expression in these tumors tended to have inferior survival outcomes, suggesting that WDHD1 probably influences patient prognosis by affecting tumor immunity.

### Genetic alterations of WDHD1 across various types of cancer

**Fig. 10:**
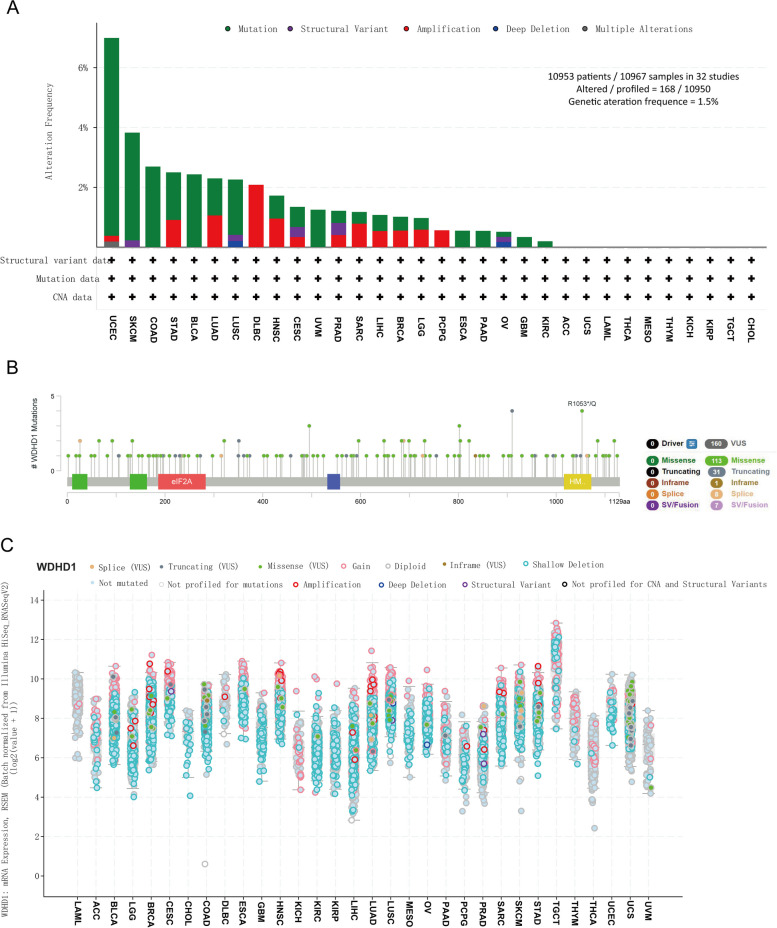
Genetic alteration of WDHD1 in pan-cancer. **A** Bar chart showing WDHD1 mutation frequencies and types in 32 cancer studies based on the TCGA PanCancer Atlas. **B** Diagram of mutation sites across WDHD1 protein domains. **C** Mutation counts and detailed types of WDHD1 in different types of cancer

Subsequently, we conducted a comprehensive genetic alteration analysis of WDHD1 in pan-cancer using the cBioPortal. The analysis included 10,953 patients out of 10,967 samples from the TCGA database, as shown in Fig. 10A. The overall mutation count of WDHD1 was examined, revealing a genetic alteration frequency of approximately 1.5%. Among the various types of cancer, patients with UCEC exhibited the highest mutation frequency of WDHD1, with “mutation” being the predominant alteration type. For types of cancer with WDHD1 alteration frequencies exceeding 2%, the primary alteration type remained “mutation,” with the exception of DLBC, where the major copy number alteration (CNA) type was “amplification.” Additionally, we identified 160 mutation sites within amino acids 0 and 1129, which included 113 missense mutations, 31 truncating mutations, one inframe mutation, eight splices, and seven structural variations (SV) or fusions. Among these, the most frequent mutation site was R1053Q (Fig. 10B). In all 32 cancers, shallow deletions of WDHD1 mRNA were common except for LAML, DLBC, KICH, THYM, THCA, and UVM (Fig. 10C).

Furthermore, we explored the genetic alterations of WDHD1 in relation to clinical outcomes in pan-cancer analysis. The analysis revealed that WDHD1 mutations were associated with improved OS (Figure S12), although the results did not reach statistical significance. A potential correlation was observed between WDHD1 mutations and OS (*p* = 0.0559, Figure S12A), DSS (*p* = 0.0778, Figure S12B), disease-free survival (*p* = 0.144, Figure S12C), and progression-free survival (*p* = 0.187, Figure S12D). These findings suggest that targeting WDHD1 mutations may hold promise as a potential approach for tumor therapy, warranting further investigation.

### Correlation of WDHD1 with tumor heterogeneity, tumor stemness, and MMR across various types of cancer

**Fig. 11:**
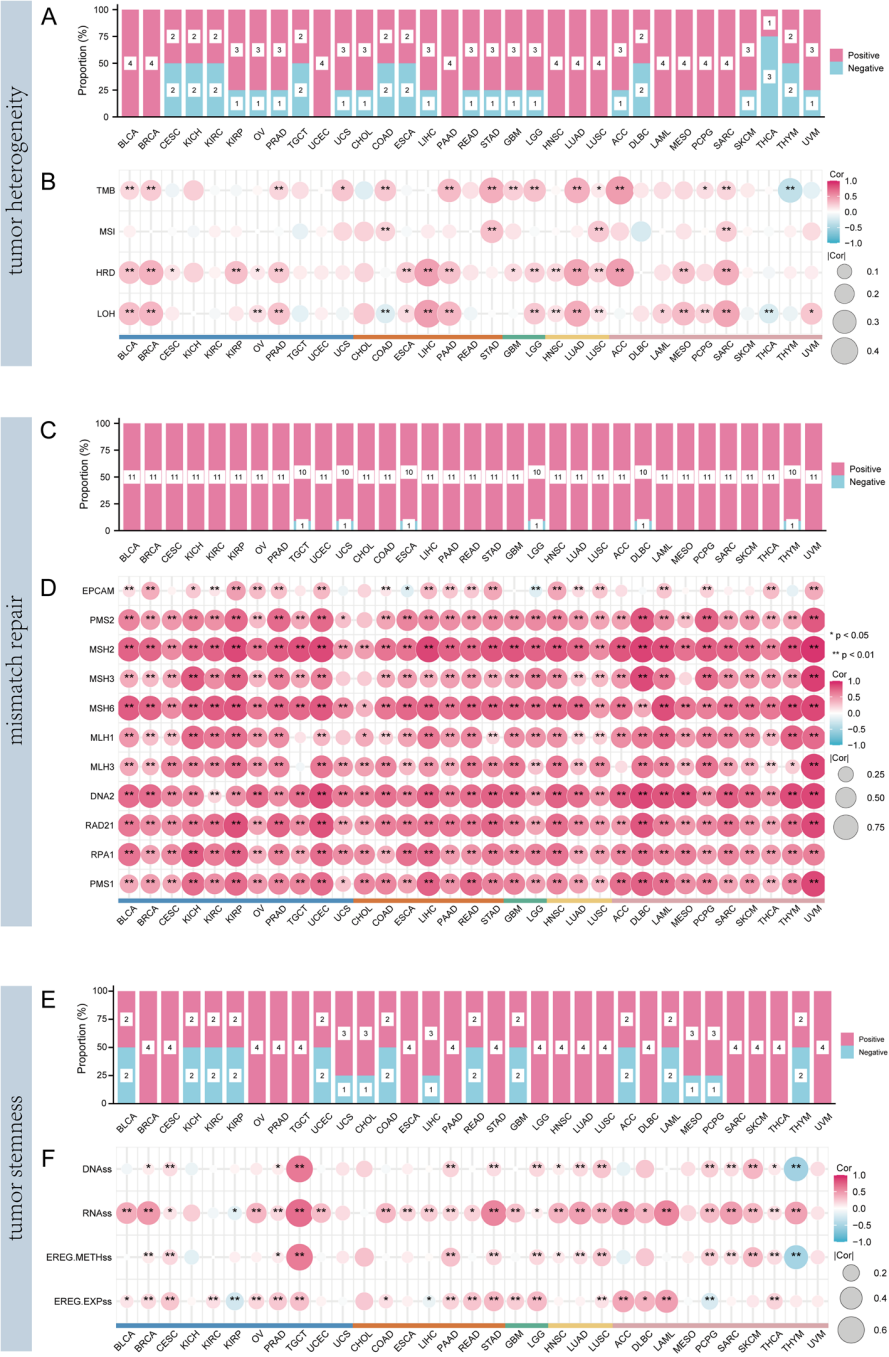
Relationship between WDHD1 expression with tumor heterogeneity, mismatch repair (MMR) genes, and tumor stemness. The bar charts show the frequency and proportion of the number of positive and negative correlations between WDHD1 and tumor heterogeneity (**A**), MMR genes (**C**), and tumor stemness (**E**). The heat map section shows the correlation between WDHD1 and specific indicators of tumor heterogeneity (**B**), MMR (**D**), and stemness (**F**). The size and color of the circles indicate the correlation coefficient. *p < 0.05, **p < 0.01

The microenvironment exerts strong and selective pressure on tumor cells, leading to their evolution. Consequently, heterogeneity arises within the same tumor, resulting in diverse populations of tumor cells [41, 42]. Tumor heterogeneity plays a significant role in tumor progression, treatment failure, and drug resistance and negatively impacts the prognosis of patients with tumors [43]. Tumor heterogeneity can be characterized by various indicators, including TMB, MSI, LOH, and HRD. TMB has long been recognized as a robust biomarker for predicting the efficacy of ICB and identifying patients eligible for immunotherapy [44]. MSI identifies defects in the DNA MMR system and is strongly correlated with immune-related objective response rates [45]. HRD leads to genomic instability and is indicative of the response to chemotherapy and polyadenosine diphosphate ribose polymerase (PARP) inhibition, particularly with platinum-based therapies [46, 47]. LOH, characterized by the loss of one allele, is an irreversible genetic alteration often associated with the loss of tumor suppressor gene function and plays a pivotal role in tumor development [48, 49]. Figure 11A shows that WDHD1 expression was significantly and positively correlated with TMB scores in 14 types of cancer. However, in THYM, a negative correlation is observed (Fig. 11A). Regarding MSI, positive correlations between WDHD1 expression and MSI scores are observed in patients with COAD, STAD, LUSC, and SARC (Fig. 11B). In the HRD analysis, positive correlations between WDHD1 expression and HRD scores were observed in 17 types of cancer (Fig. 11B). Similarly, positive and significant correlations between WDHD1 and LOH scores can be seen in 16 types of cancer. However, in COAD and THCA, WDHD1 and LOH scores exhibit a negative association (Fig. 11B). Figure 11A shows the frequency and proportion of correlations between WDHD1 and the four heterogeneity indicators for each cancer. Notably, in LUSC and SARC, WDHD1 shows significant positive correlations with all four indicators. The association between WDHD1 and MMR genes was also evaluated, and the results suggest a positive and significant correlation in more than 30 tumors; they were positively and significantly correlated (Fig. 11C, D). In summary, in most cases, WDHD1 exhibits a positive correlation with tumor heterogeneity, suggesting its potential influence on tumor heterogeneity and promotion of tumor progression.

Cancer stem cells (CSCs) are capable of self-renewing, and they initiate tumors, sustain tumor growth, maintain the tumor microenvironment (TME), promote tumor metastasis, and induce drug-therapy resistance [50, 51]. The stemness indexes measure how similar tumor cells are to stem cells. Four stemness indexes were incorporated into our study based on mRNA expression and DNA methylation, respectively. The stemness indexes ranged between 0 and 1, with 0 indicating that tumor cells had less similarity to stem cells and 1 indicating that more remarkable similarity existed between tumor cells and stem cells. The association between WDHD1 and cancer stemness indexes across various types of cancer was then examined. We found that WDHD1 expression in most types of malignancy was significantly correlated with cancer stemness indexes, revealing a close relationship between WDHD1 and tumor stemness formation (Fig. 11E, F).

### Correlations of WDHD1 and RNA methylation modifications across various types of cancer

**Fig. 12:**
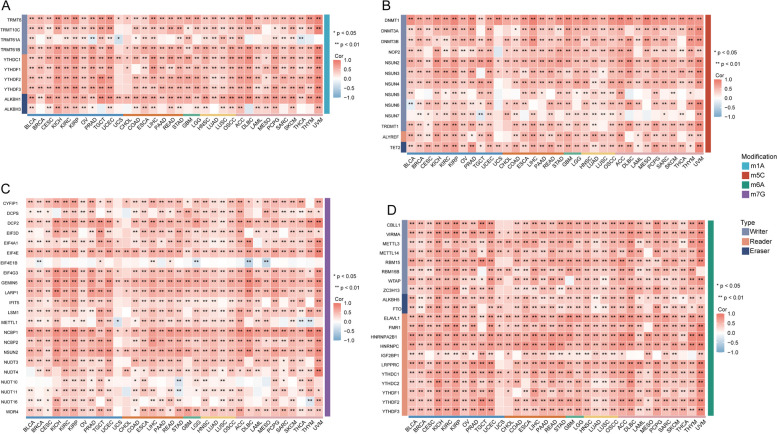
Relationship between WDHD1 expression with RNA methylation modifications regulators in pan-cancer. Heatmap illustrating the correlation between WDHD1 and m1A regulatory genes (**A**), m5C regulatory genes (**B**), m7G regulatory genes (**C**), and m6A regulatory genes (**D**) (*p < 0.05, **p < 0.01)

RNA methylation plays a crucial role in the regulation of various biological processes, including RNA transcription, stability, and translation. It has been extensively documented that dysregulation of RNA methylation contributes to the development and progression of various human cancers [52]. To further investigate the tumorigenic roles of WDHD1, we analyzed the associations between WDHD1 and regulators of N1-methyladensoine (m1A) methylation, 5-methylcytosine (m5C) methylation, N7-methylguanosine (m7G) methylation, and N6-methyladenosine (m6A) methylation. The results revealed a positive correlation between WDHD1 and m1A regulators in most cancers, except for CHOL and UCS (Fig. 12A). Similarly, significant positive associations between WDHD1 and m5C regulators were observed in pan-cancer analyses. Notably, DNMT1, NSUN2, and TET2 showed consistent positive correlations with WDHD1 expression across all types of cancers (Fig. 12B). The correlations between WDHD1 and m7G regulators were least significant in CHOL and UCS (Fig. 12C). Moreover, a multitude of m6A regulators exhibited strong positive correlations with WDHD1 expression across different types of cancer (Fig. 12D). Overall, WDHD1 showed significant and positive correlations with a majority of RNA methylation regulators across various types of cancer, suggesting its potential role in regulating the RNA methylation process and influencing tumorigenesis.

### The functional state of WDHD1 as identified by single-cell RNA sequencing data

**Fig. 13:**
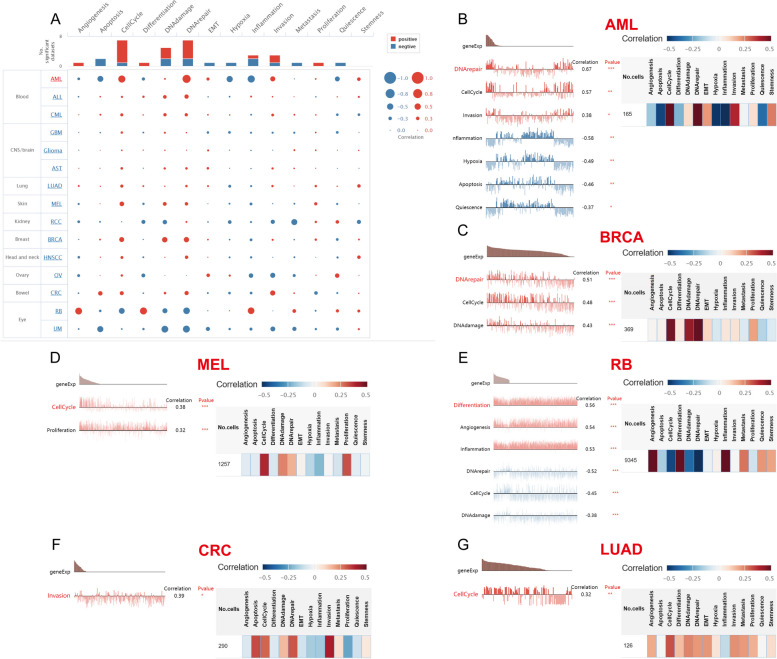
Correlation of WDHD1 with functional states in CancerSEA datasets. **A** Role of WDHD1 in cancer for different functional states as represented by an interactive bubble chart. The significant correlations of WDHD1 with functional states in AML (**B**), BRCA (**C**), MEL (**D**), RB (**E**), CRC (**F**), and LUAD (**G**) are displayed

To gain insights into the functional state of WDHD1 at the single-cell level in specific cancers, we utilized CancerSEA. This allowed us to examine the correlation of WDHD1 with multiple aspects of cancer cells. As shown in Fig. 13A, WDHD1 exhibited a relatively strong and positive association with cell cycle regulation, DNA damage and repair, and invasion. The correlation between WDHD1 and angiogenesis, differentiation, and proliferation was relatively weak but still positive. Conversely, the associations between WDHD1 and apoptosis, EMT, hypoxia, inflammation, metastasis, and quiescence were predominantly negative, with some associations showing weaker correlations (Fig. 13A). Furthermore, we explored the relationship between WDHD1 and functional status in specific types of cancer. In AML, WDHD1 is positively correlated with DNA repair, cell cycle regulation, and invasion. In BRCA, WDHD1 showed positive correlations with DNA repair, cell cycle regulation, and DNA damage. It exhibited a positive correlation with cell cycle and proliferation in melanoma (MEL), differentiation, angiogenesis, and inflammation in retinoblastoma (RB), invasion in colorectal cancer (CRC), and cell cycle regulation in LUAD. Conversely, in AML, WDHD1 was negatively correlated with inflammation, hypoxia, apoptosis, and quiescence, and in RB, it was negatively correlated with DNA repair, cell cycle regulation, and DNA damage (Fig. 13B–G).

**Fig. 14:**
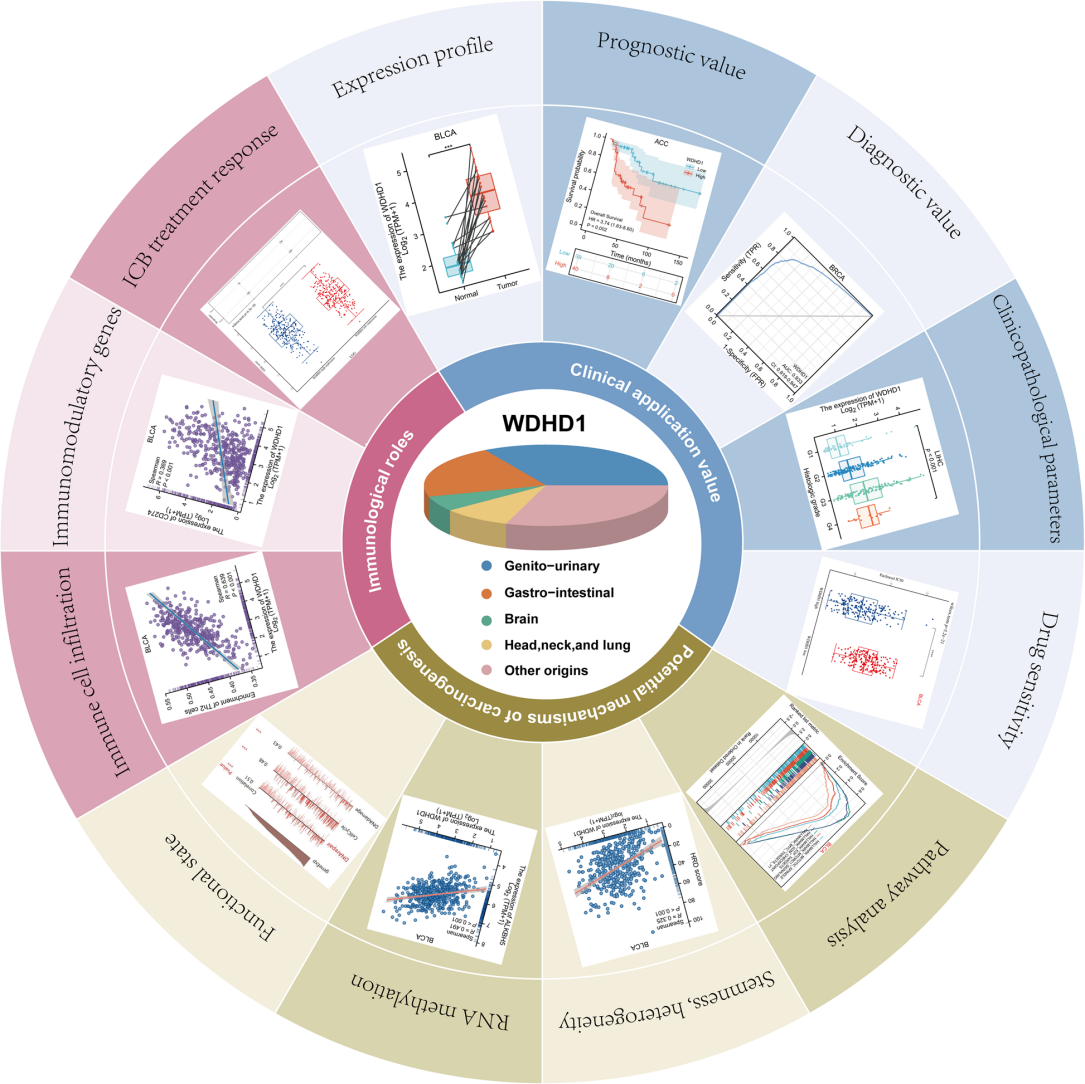
A brief summary of the main findings in this work. WDHD1 is highly expressed in tumor tissues and is a potential pan-cancer prognostic and diagnostic biomarker. WDHD1 expression is associated with tumor grading and staging and correlates with the IC50 of rapamycin and paclitaxel. Meanwhile, WDHD1 is linked to many oncogenic pathways and may participate in tumorigenesis through influencing tumor heterogeneity, stemness, and RNA methylation modifications. In the final, WDHD1 is associated with the TIME and can predict the efficacy of immunotherapy

## Discussion

DNA replication in eukaryotic cells is facilitated by a complex machinery known as the replisome, which consists of the replisome progression complex (RPC) and DNA polymerases [53, 54]. The RPC includes a highly conserved protein called WDHD1, with orthologs found in fungi and vertebrates. In yeast, a WDHD1 homolog called CTF4 has been identified and shown to be crucial for maintaining genomic integrity and modulating DNA damage repair and telomere replication processes [55, 56]. WDHD1 is a DNA-binding protein located in the nucleus and cytoplasm, and it contains several functional domains. Notably, the N-terminal region contains the WD40 domain [57], which is essential for cell proliferation, genome integrity maintenance, signal transduction, and cytoskeleton assembly [17, 54]. The central region of WDHD1 contains the SepB domain responsible for its interaction with DNA polymerase α [58]. The C-terminal region of WDHD1 contains the high mobility group box (HMB) domain, which has been implicated in chromatin assembly, DNA replication, post-transcriptional regulation of the centromeric pathway, and the formation of nucleoprotein complexes [9, 59]. Recent studies have revealed the interaction between WDHD1 and MCM10, highlighting its role as an important factor for replication initiation [55]. Depletion of WDHD1 using siRNA has been shown to impede proliferation in human cells by causing a delay in cell cycle progression from late S phase to G2 [60]. Similar effects have been observed in mice, where disruption of WDHD1 decreased proliferation and induced apoptosis in mouse embryos [61]. Furthermore, recent speculations have arisen regarding the involvement of WDHD1 in G1 checkpoint control [62]. Collectively, these findings suggest that WDHD1 plays an essential role in initiating DNA replication and regulating cell proliferation in normal cells.

However, research on the mechanism of WDHD1 in cancer has not been extensive. Several studies have linked the WDHD1 gene to key processes such as tumorigenesis and metastasis [18, 63, 64]. For example, Li et al. proposed that WDHD1 could regulate the stability of the GCN5 protein and the acetylation of histone H3 with remarkable precision, which might play an important role in tumor cell growth [65]. As validated by bioinformatics and cellular experiments, respectively, Chen et al. and Liu et al. found that WDHD1 was associated with lymph node metastasis in CESC and CHOL [63, 66]. Furthermore, several studies have shown that WDHD1 is associated with the regulation of tumor cell stemness, chemoresistance, and cell cycle regulation [13, 16, 67]. Of particular note, Ertay et al. proposed that WDHD1 may control stemness of phosphatase and tensin homolog (PTEN)-inactive triple-negative breast cancer (TNBC) cells through its ability to regulate and protein translation [16]. Consistently, we also found that WDHD1 is associated with stemness in many tumors, including BRCA, CESC, and PRAD. Besides, we delineated the genomic features of WDHD1 at the pan-cancer level, including expression and mutations, and explored for the first time the relationship between its paclitaxel and rapamycin resistance, tumor heterogeneity, RNA methylation modification, MYC, and E2F pathways. We also proposed hypotheses that WDHD1 may promote tumor immune escape and that WDHD1 mutations are potential tumor therapeutic options. Considering that some studies have also recently discovered potent inhibitors of WDHD1, which provide a new approach to the treatment of various cancers, our study may provide more comprehensive clues to further understand the mechanism of the role of WDHD1 in tumors and provide new references for the development of new tumor therapeutic strategies [68].

The initial step of this study involved examining WDHD1 expression and its diagnostic value in multiple cancers. Our findings revealed consistent WDHD1 overexpression in tumors at both mRNA and protein levels compared to normal tissues. This is consistent with previous studies that have demonstrated elevated WDHD1 mRNA expression in various types of cancer, including LUAD, esophageal cancer, CHOL, colorectal cancer, and laryngeal squamous cell carcinoma (LSCC), while showing no significant expression in normal tissues [13, 18, 65, 68–70]. Our expression analysis results were consistent with these previous findings. Additionally, researchers have also observed a higher WDHD1 expression in PTEN-inactive TNBC cells, human papillomavirus (HPV)-16 oncogene E7-expressing spontaneously immortalized human foreskin keratinocytes, cholangiocarcinoma cell lines, and MEL cells [17, 18, 63, 65]. Notably, Wu et al. conducted a diagnostic study on LSCC and concluded that WDHD1 could serve as a robust biomarker to distinguish between LSCC and non-LSCC, with AUC values of ROC curves usually exceeding 0.75 [70]. In our ROC analysis, we found AUC exceeding 0.7 in 27 cancers and 0.9 in 16 cancers, indicating that WDHD1 could effectively distinguish between tumorous and corresponding normal tissues. These observations highlight the crucial role of increased WDHD1 expression in tumor development and suggest its potential as a pan-cancer diagnostic biomarker in the future. However, the use of WDHD1 as a potential diagnostic biomarker may have the following limitations and challenges in the future. First, although our study found that WDHD1 mRNA has high diagnostic value in many tumor types and provided cutoff values for the ROC curves of each tumor, the predictive performance of the ROC curves varies with the environment [71]. In future clinical management, it is debatable how to determine the cutoff value to make WDHD1 perform the best diagnostic value in different regions and different tumor types. In addition, how to select the most appropriate specimens for testing (for example, blood samples), and are there advantages of this approach over traditional diagnostic methods such as pathology and imaging [72]? Furthermore, can WDHD1 be widely used in clinical applications like the current commonly used cancer biomarkers such as CA199 and CA125 [73]? Finally, since we only explored the diagnostic value of WDHD1 mRNA, what would be the diagnostic value of evaluating its protein level using proteomics technology [74]? The above limitations and challenges need to be confirmed by subsequent basic and pre-clinical studies.

Our survival analysis based on the TCGA cohort revealed that WDHD1 serves as a prognostic biomarker in pan-cancer analysis. High WDHD1 expression was associated with poor prognosis in cancers originating from the gastrointestinal (LIHC and PAAD), lung (LUAD), brain (LGG), and tumors of other origins (ACC, MESO, and SARC), as evidenced by OS, DSS, and PFI. Additionally, increased WDHD1 expression was associated with shorter DSS and PFI in BLCA, shorter OS and PFI in KICH, and shorter OS and DSS in KIRP and SKCM. However, WDHD1 was identified as a protective factor in OS analysis for KIRC, READ, and THYM. To further validate these findings, we analyzed survival information from various GEO datasets. The results showed that, in addition to the aforementioned cancers, high WDHD1 expression was also associated with inferior survival outcomes in patients with BRCA and blood cancers. Collectively, these results suggest that WDHD1 holds prognostic value across tumors originating from different organs. Additionally, WDHD1 has been implicated in the malignant behavior of certain tumors. For instance, in cervical cancer, WDHD1 has been identified as a critical gene associated with lymph node metastasis. Knockdown of WDHD1 in cervical cancer cells expressing the E7 oncogene resulted in a significant decrease in cell cycle proliferation and DNA synthesis, thereby hindering HPV-induced carcinogenesis [64, 66].

For breast cancer, it has been reported that WDHD1 plays a crucial role in sustaining the survival of PTEN-inactive TNBC cells by mediating a high demand for protein translation [16]. Moreover, Sato et al. have demonstrated that patients with positive WDHD1 immunostaining in non-small-cell lung cancer (NSCLC) and esophageal cell squamous carcinoma had poor prognoses. Their subsequent cell experiment illustrated that WDHD1 acted as a pivotal cell cycle regulator and an essential component of the phosphoinositide-3-kinase-protein kinase B (PI3K)/Akt pathway. Similar conclusions were reached by Xian et al. who suggested that WDHD1 might be an irreplaceable tumor factor in the case of esophageal cancer [18, 69]. Concerning CHOL, inhibiting WDHD1 repressed cell migration and invasion while promoting cell apoptosis [63]. In laryngeal cancer, high WDHD1 expression was observed in patients with lymph node metastases or higher stages [70]. For AML, a study found that knocking down WDHD1 impaired cell viability and growth [75]. Notably, in our study, we reported for the first time that WDHD1 expression was associated with higher clinical grading and staging in LIHC and UCEC. Previous studies have shown that WDHD1 is closely related to the maintenance of genomic integrity and the DNA replication process, and genomic instability accelerates the acquisition of genetic diversity [76]. Meanwhile, the uncontrolled replication process provides tumor cells with an inexhaustible source of fuel [77]. They are both hallmarks of cancer and are closely associated with tumor progression. This may partially explain the involvement of WDHD1 in the progression of UCEC and LIHC. In particular, WDHD1 has been reported to be associated with the transcriptional activity of HPV-related genes [64]. Also, as a tumor closely associated with viral infection, the relationship between WDHD1 and HBV infection in LIHC deserves to be explored in depth. In addition, WDHD1 has also been reported to be associated with centromeric epigenetic modifications, which in turn lead to inactivation of some tumor suppressor genes or activation of oncogenes, promoting tumor progression [59]. Of course, our current findings are also indicative for the clinical management of LIHC and UCEC, such as the design of kits for the diagnosis of early-stage endometrial and hepatocellular carcinomas (containing primers, probes, or antibodies targeting the WDHD1 gene), which will lead to a more accurate and rapid diagnosis of the two tumors. In addition, considering the close relationship between WDHD1 and DNA repair pathways, mechanistic studies of WDHD1 resistance to chemotherapy and radiotherapy in these two tumors are equally attractive. Finally, with the development of artificial intelligence and machine learning algorithms, we can better understand the mechanism of action of WDHD1, so as to optimize the clinical management of tumor patients and determine the appropriate treatment plan for them, thus improving the clinical success rate [78].

Apart from the above-stated functions that include cell cycle regulation and tumor growth stimulation, WDHD1 also contributed to cisplatin resistance in LUAD and was identified as a radiosensitization target in NSCLC [13, 79]. Collectively, these findings highlight the importance of WDHD1 in cancer cell cycle progression and patient outcomes. In our study, we explored the association between WDHD1 expression and prognosis in various cancers and found a negative correlation. Notably, in genito-urinary (BLCA, KICH, and KIRP), gastrointestinal (LIHC and PAAD), and brain (LGG) tumors, as well as tumors of other origins (ACC, MESO, SARC, and SKCM), WDHD1 exhibited good prognostic value, although there were scarce reports explaining its specific role in these tumors. Additionally, our study on the association between WDHD1 and clinicopathological parameters showed that WDHD1 expression increased with the advancing staging of KICH, KIRP, UCEC, LIHC, LUSC, ACC, and grading of BLCA, UCEC, PAAD, LIHC, HNSC, and LGG. These findings suggest the possible involvement of WDHD1 in the progression of these tumors. In summary, our study provides a pre-existing basis for studying WDHD1 in a broader range of different types of cancer, offering insights into its potential as a prognostic marker and therapeutic target.

The GO enrichment analysis conducted in our study revealed that WDHD1 is involved in the BP and MF related to DNA replication and repair. Moreover, it plays a role in various cellular components (CCs), including chromosomal structures, centromeres, and replication forks. Our KEGG analysis further elucidated a close relationship between WDHD1 and carcinogenesis pathways, such as cell cycle regulation, homologous recombination, the mismatch pathway, and DNA replication. These results were corroborated by our single-cell functional analysis. It is widely accepted that cells heavily rely on a well-regulated cell cycle to ensure accurate DNA replication and precise cell division, which are crucial for the faithful transmission of genetic information [80, 81]. Tumorigenesis is primarily driven by aberrant cell cycle progression, enhanced DNA replication, and subsequent deregulated cell proliferation [82–85]. Studies have reported that WDHD1 governs the assembly of MCM2-7 at the replication region during late mitosis and the early G1 phase, indicating its important role in regulating pre-replication complex (RC) assembly [56]. Another critical function of WDHD1 is to maintain the stability of polymerase *α* and facilitate its loading at replication origins [55, 86]. Additionally, there are supporting studies suggesting that WDHD1 acts as a regulator of the G1 checkpoint [17, 62]. Notably, our GESA demonstrated that the G2/M checkpoint was enriched in the WDHD1-high phenotype, indicating that WDHD1 might broadly regulate checkpoints in the cell cycle. Lastly, it is essential for normal cells to maintain genomic integrity under conditions of continuous exposure to endogenous and exogenous DNA damage [87]. The deficiency in DNA damage repair leads to genomic instability, which is a well-established contributor to tumor initiation and progression and is considered a hallmark of cancer [88–90]. The *WDHD1* gene plays a critical role in regulating chromatin structure, facilitating genome stability, and enabling efficient DNA synthesis [91, 92]. Depletion of WDHD1 affects DNA-end resection, impairs homologous recombination repair, and interferes with the maintenance of DNA damage checkpoints [91]. Our study observed a positive correlation between WDHD1 and MMR gene expressions, confirming its involvement in the DNA repair pathway and supporting the findings of Li et al. who reported that *WDHD1* binds to *MSH2* and contributes to the MMR pathway [93]. Considering these observations alongside the frequent WDHD1 overexpression in the aforementioned cancers, we speculate that dysregulation of the function of WDHD1 in DNA replication, cell cycle regulation, and DNA damage repair may contribute to its oncogenic potential.

According to GSEA, high WDHD1 expression was predominantly associated with hallmark gene sets, including MYC targets, the mitotic spindle, mTORC1 signaling, E2F targets, and the G2/M checkpoint. In oncogenic signatures, high WDHD1 expression correlated with MYC, mTOR, and E2F, whereas low WDHD1 expression was associated with p53, a tumor-suppressive signature. The regulatory roles of MYC, mTOR, and E2F in tumor initiation and progression processes are widely recognized. In many cancers, mTOR exhibits hyperactivity and functions as an effector downstream of various oncogenic pathways, such as PI3K/Akt and Ras/Raf/Mek/Erk (MAPK) [94]. Upregulated mTOR signaling promotes tumor growth by enhancing the signaling of growth factor receptors, facilitating migration, angiogenesis, metabolism, and suppressing autophagy [94–96].

E2F plays a crucial role in cell division, chromosome stability, and DNA damage response, which aligns closely with the physiological function of WDHD1 [97, 98]. Dysregulated E2F activity can lead to the failure of tumor cells to respond to cell cycle exit signals, partly mediated by the deregulation of its upstream regulator, the RB protein [99, 100]. Elevated E2F expression in patients with tumors has been associated with poorer survival outcomes [98]. The oncogene MYC is frequently amplified and is considered a downstream effector of various oncogenic signaling pathways in human cancers [34, 101]. It plays a pivotal role in tumorigenesis and cellular metabolic activities by triggering selective gene expression amplification, ultimately promoting growth and proliferation [102, 103]. Suppressing MYC expression or inhibiting its function has been shown to lead to significant tumor regression [101, 104, 105]. Notably, activation of MYC induces the activation of E2F transcription factors, promoting cell cycle progression into the S phase, suggesting a potential synergistic effect between E2F and MYC [106]. Moreover, MYC-induced carcinogenesis has been associated with mutant *TP53* [107]. These findings are consistent with the GSEA results in our study. However, despite these observations, a comprehensive literature review revealed no studies that extensively explored the relationship between WDHD1 and the abovementioned pathways. Therefore, to elucidate the specific oncogenic mechanism of WDHD1, it is essential to conduct detailed investigations into the interplay between WDHD1 and these pathways in the future.

Based on the description above, WDHD1 appears to be closely associated with mTOR signaling, the mitotic spindle, and cell replication. To provide more relevant clinical insights, we studied the association between WDHD1 expression and the IC_50_ values of two commonly used chemotherapy drugs, rapamycin and paclitaxel. Rapamycin and its derivatives, known as rapalogs, belong to the first generation of mTOR inhibitors that effectively target mTORC1 by binding to FKBP-12 and forming a ternary complex [108]. These drugs have shown promising results in inhibiting tumor growth by inducing cell cycle delay, promoting apoptosis, and inhibiting oncogenic transformation in human tumor cells [95, 109]. Furthermore, in in vivo mouse models, rapamycin showed the ability to inhibit metastatic tumor growth and suppress angiogenesis [110]. Paclitaxel, another potential anticancer drug, exerts its effects by inhibiting the cell cycle at the G2/M phase, inducing apoptosis, and preventing cell replication [111]. Mechanistically, paclitaxel disrupts normal microtubule dynamics, a critical process for cell division and interphase events, thereby impeding cell cycle progression, blocking mitosis, and effectively eliminating tumor cells [112–114]. Specifically, paclitaxel enhances and stabilizes the binding of stable microtubules, crucial for the formation of mitotic spindles [111]. Despite the remarkable efficacy of rapamycin and paclitaxel in cancer treatment, their non-specific toxicity towards healthy tissues, including hair follicles, bone marrow, and gastrointestinal tract cells, can result in severe side effects that should not be overlooked [111]. Therefore, it is essential to identify individuals who are sensitive to these chemotherapy drugs, allowing for the minimization of adverse effects. In our study, we discovered that patients with high WDHD1 expression in BRCA, KIRP, and LIHC and low WDHD1 expression in ACC were more sensitive to rapamycin treatment (Fig. 7A). Furthermore, we observed that patients with high WDHD1 expression in all ten studied cancers were more sensitive to paclitaxel treatment (Fig. 7B). These results indicate that WDHD1 could serve as a potential biomarker for predicting the response to rapamycin and paclitaxel treatment in specific cancers, which could help in avoiding the unwanted toxic side effects associated with chemotherapy drugs.

Given the aforementioned diversity of WDHD1 functions, we propose the following potential tumor therapeutic strategies for targeting WDHD1. First, potential disruptors targeting the interactions between WDHD1 and other members of its protein complex (*Tipin* and *Tim1*) could be used as therapeutic approaches for WDHD1-amplified cancers [11]. Second, inhibition of WDHD1 function by gene editing techniques, such as CRISPR-Cas9 [115]. Third, considering the importance of WDHD1 to the DNA damage repair process, inhibition of WDHD1 may be effective in increasing the sensitivity of radiotherapy and chemotherapeutic agents and overcoming resistance to these treatments due to increased homologous mismatch repair [68]. Fourth, targeting these potential pathways of action of WDHD1, such as the E2F and MYC pathways. This enables the application of inhibitors of these pathways in a subgroup of WDHD1-active tumors. Of course, WDHD1-targeted therapies may still have the following difficulties and challenges. To begin with, we still lack sufficient understanding of the structure and appropriate active sites of the WDHD1 protein, which makes it even more difficult to find suitable targeting drugs [116]. Moreover, since WDHD1 is also expressed in normal tissues, how to design highly selective therapeutic approaches to maximize efficacy and minimize toxicity to achieve precision targeting also remains to be addressed. Finally, targeting WDHD1 to treat cancer patients with different molecular combinations may not be optimal, considering that advanced tumors usually contain several molecularly altered and rapidly evolving subclones [117]. Of course, due to the different tumor sites that need to be acted upon (e.g., brain tumors), physiological barriers such as the blood–brain barrier may exist, and it is also worth exploring how to overcome these absorption barriers and explore more appropriate delivery modes so that these drugs can better reach the target organs and exert their bioavailability at a higher level [118]. Despite the above difficulties and challenges in targeting WDHD1 therapies, however, we believe that with the continuous development of precision medicine and the growing understanding of the association of WDHD1 biology with cancer, more WDHD1 inhibitors will be integrated into a mechanistically richer and larger pipeline of targeted drugs.

In the microenvironment surrounding solid tumors, a large number of immune cells and non-immune stromal cells infiltrate, and their presence is closely associated with patient clinical outcomes [119]. Our study has revealed that WDHD1 expression is negatively associated with stromal score and immune score in various types of cancer. Additionally, there is a negative correlation between WDHD1 and the infiltration of several immune cells, including CD8 + T cells, cytotoxic cells, dendritic cells (DCs), and plasmacytoid DCs (pDCs), all of which play crucial roles in the body’s anti-tumor immunity. CD8 + T cells are recognized as major anticancer effector cells due to their ability to generate cytotoxic T lymphocytes that target and eliminate tumorigenic cells by recognizing peptide MHCs [119, 120]. Cytotoxic cells also exhibit cytotoxicity against cancer cells and mediate durable and efficient anti-tumor immune responses [120–122]. DCs, on the other hand, are highly efficient at inducing antigen-specific T cell responses, making them one of the most important antigen-presenting cells and essential for eliciting potent and powerful anti-tumor immunity [123, 124].

Moreover, our findings revealed a positive correlation between WDHD1 expression and Th2 cell infiltration in all cancers except CHOL, which is particularly noteworthy. In the TME, cancer cells are typically targeted by Th1 cells, which provide crucial assistance to CD8 + T cells and stimulate the tumoricidal activity of macrophages [125]. On the other hand, Th2 cells secrete cytokines that promote a tumor-promoting M2-like phenotype in macrophages associated with tumors and facilitate tumor immune evasion within the TME [126]. The balance between Th1 and Th2 responses plays a significant immunoregulatory role and directly impacts tumor progression [127]. Generally, a shift in favor of the Th2 response contributes to pro-tumorigenic consequences within the TME and is associated with poorer prognoses in patients with cancer [128]. Considering these findings, it is reasonable to infer that WDHD1 may play a role in promoting immune escape by negatively correlating with several immunological effector cells, thereby tilting the balance toward an immunosuppressive TME. In the context of future clinical trials, investigating the potential use of WDHD1 as a biomarker for identifying the Th1/Th2 immune response status of patients with tumors could be a promising avenue.

Additionally, we discovered a correlation between WDHD1 expression and immunoregulatory genes across various types of cancer. We focused particularly on five cancers in which WDHD1 showed the strongest association with immunoinhibitors. Patients with high WDHD1 expression in LGG, LIHC, LUAD, or PAAD demonstrated higher TIDE scores compared to those with low WDHD1 expression. The TIDE algorithm is a commonly used tool for assessing the immune escape potential of the tumor and predicting the response to ICB therapy. Our findings indicate that high WDHD1 expression is associated with immunosuppression and potentially poorer immunotherapeutic efficacy in these four types of tumors. Numerous studies have documented that the activation of immunoinhibitors on the surface of tumor cells enables them to evade immune surveillance [129, 130]. Mechanistically, immunoinhibitors such as cytotoxic T lymphocyte-associated molecule-4 (CTLA-4), programmed cell death receptor-1 (PD-1), and PD ligand-1 (PD-L1) act as direct negative regulators of effector cytotoxic cells, inducing immune tolerance and inhibiting anti-tumor immune responses [131, 132]. Thus, the positive correlations observed between WDHD1 and various immune checkpoints in our study may suggest that WDHD1 promotes immune escape through another mechanism, potentially by modulating the expression or activity of these immunoinhibitory molecules. Moreover, recent research has proposed an association between the cell cycle and immune escape, and ICB’s antitumor effect could be enhanced by therapeutic approaches targeting cell cycle regulators [83]. Although WDHD1-based immunotherapy is promising, we predict that the following challenges may exist. First, although we used WDHD1 expression TIDE values to screen for populations more likely to benefit from immune checkpoint therapy, given the complexity of the tumors and the heterogeneity of the population, future consideration should be given to the joint application of additional metrics, such as tobacco exposure and hepatitis B virus infection, to further identify populations that are most likely to benefit from treatment with ICIs across different tumor types, as these factors are also associated with the lack of use of tumor of immunotherapy outcomes [133–135]. Second, rational biomarker-based combination therapies may be an option for achieving long-term efficacy in patients with tumors. However, the toxicity of such combination therapies needs to be considered, and subsequent prospective studies may be needed to link histological profiles and immunophenotypes with efficacy data to better identify immunotherapies that can be transitioned to clinical trials and to identify and manage adverse events early to provide truly personalized precision medicine for patients [133, 136].

Given our previous discussion on the association of WDHD1 with the cell cycle, we hypothesize that targeting WDHD1 could serve a dual purpose in anticancer therapy, disrupting cancer cell division while restoring cancer immune surveillance. This approach may offer a potential “two birds with one stone” strategy in cancer treatment. Furthermore, there is growing evidence supporting the notion that paclitaxel can boost antitumor immunogenicity and stimulate the immune system to more effectively attack tumors [137–139]. In our study, we observed that patients with high WDHD1 expression showed a higher sensitivity to paclitaxel. Considering the success of the combined use of atezolizumab plus paclitaxel in metastatic TNBC, the combination of paclitaxel and immunotherapy in patients with high WDHD1 expression holds promise for enhancing the efficacy of cancer treatment [139]. However, rigorous clinical trials are essential to verifying these potential benefits. Despite our significant findings, our study does have some limitations. The data used in our study were mainly gathered from public databases, and the quality of the data may need further confirmation. Additionally, to validate our findings, future in vivo and in vitro experiments are required.

## Conclusion

Currently, there is a growing interest in pan-cancer research, aiming to gain deeper insights into tumor initiation and development. In our study, we comprehensively investigated the multifaceted roles of WDHD1 in pan-cancer research. As shown in Fig. 14, we observed that WDHD1 was significantly overexpressed in the majority of tumor tissues, highlighting its potential as both a prognostic and diagnostic biomarker. Through functional enrichment analyses, we identified WDHD1 as a key participant in cell cycle regulation and DNA damage repair processes while also being closely associated with several pathways associated with cancer, including E2F, MYC, and mTOR signaling. Additionally, WDHD1 expression showed correlations with the drug sensitivity of rapamycin and paclitaxel in various types of cancer. Our subsequent immune-relevant analysis demonstrated that WDHD1 expression was associated with immune cell infiltration and immunomodulatory molecule expression. Notably, the use of WDHD1 as a predictive marker for the ICB response in specific cancers emerged as a possibility from our study. Furthermore, our investigations delved into the close relationship between WDHD1 and tumor heterogeneity, DNA MMR, tumor stemness, and RNA methylation modifications, suggesting the involvement of WDHD1 in numerous other carcinogenic processes. These findings collectively highlight the significance of WDHD1 in tumorigenesis and TME, potentially facilitating the clinical application of WDHD1-based therapies.

### Supplementary Information

**Additional file 1:** **Figure S1.** WDHD1 mRNA expression between tumor and normal tissues in 20 independent cohorts from the GEO database. T is short for tumor tissues, and N is short for normal tissues (* *p *< 0.05, ** *p* < 0.01, *** p < 0.001). **Figure S2.** WDHD1 mRNA expression between tumor and normal tissues in additional 22 independent cohorts from the GEO database (* p < 0.05, ** p < 0.01, *** *p* < 0.001). **Figure S3.** WDHD1 protein expression between normal and tumor tissues by the UALCAN (**** p < 0.0001, ns, not statistically significant). **Figure S4.** The ROC curves indicate that WDHD1 has an excellent diagnostic value in the TCGA pan-cancer cohort. The true positive rate (TPR) is shown on the Y-axis and the false positive rate (FPR) is shown on the X-axis. Diagnostic accuracy increases with a larger area under the curve (AUC). **Figure S5.** The diagnostic value of WDHD1 was evaluated using the GEO dataset (41 independent cohorts in total) as external validation. **Figure S6.** The relationship between WDHD1 and disease-specific survival (DSS). (A) A DSS forest plot of the pan-cancer cohort. Tumors are arranged according to different origins of tissue (color distinction). The association between WDHD1 expression and patient DSS in KIRP (B), BLCA (C), LIHC (D), PAAD (E), LGG (F), LUAD (G), ACC (H), MESO (I), SARC (J), and SKCM (K) is analyzed using Kaplan-Meier methods. **Figure S7.** The relationship between WDHD1 and progression-free interval (PFI). (A) A PFI forest plot of the pan-cancer cohort. The association between WDHD1 expression and patient PFI in KICH (B), PRAD (C), BLCA (D), OV (E), PAAD (F), LIHC (G), LGG (H), GBM (I), LUAD (J), ACC (K), PCPG (L), MESO (M), and SARC (N) is analyzed using Kaplan-Meier methods. **Figure S8.** WDHD1 survival analysis using 26 independent cohorts from the GEO datasets. In most cases, patient with high WDHD1 expression has a significant worse prognosis. **Figure S9.** Survival analysis of WDHD1 from the PrognoScan database. A total of 16 independent cohorts are included in the research. **Figure S10.** Survival analysis of WDHD1 from the PrognoScan database. Additional 11 independent cohorts are included in the research. **Figure S11.** TIDE score of WDHD1 high and low expression groups in BLCA (A), LGG (B), LIHC (C), LUAD (D), and PAAD (E). * *p* <0.05, **** p < 0.0001, ns, not statistically significant. **Figure S12.** The potential correlation between the mutation status of WDHD1 and overall survival (OS) (A), disease-specific survival (DSS) (B), disease-free survival (DFS) (C), and progression-free survival (PFS) (D) of the TCGA PanCancer cohort.

**Additional file 2: Table S1.** The results of the PH assumption before Cox regression analysis. **Table S2.** Detailed information on ROC curves for TCGA and GEO cohorts.

## Data Availability

This study analyzed publicly available datasets. These datasets are available from the TCGA (https://portal.gdc.cancer.gov/) and GEO databases (https://www.ncbi.nlm.nih.gov/geo/).
