# Supplementary figures and images for: Integrative bioinformatics analysis of WDHD1: a potential biomarker for pan-cancer prognosis, diagnosis, and immunotherapy
Source: World J Surg Oncol. 2023 Sep 27;21:309. doi: 10.1186/s12957-023-03187-3 (PMC10523704; doi:10.1186/s12957-023-03187-3)

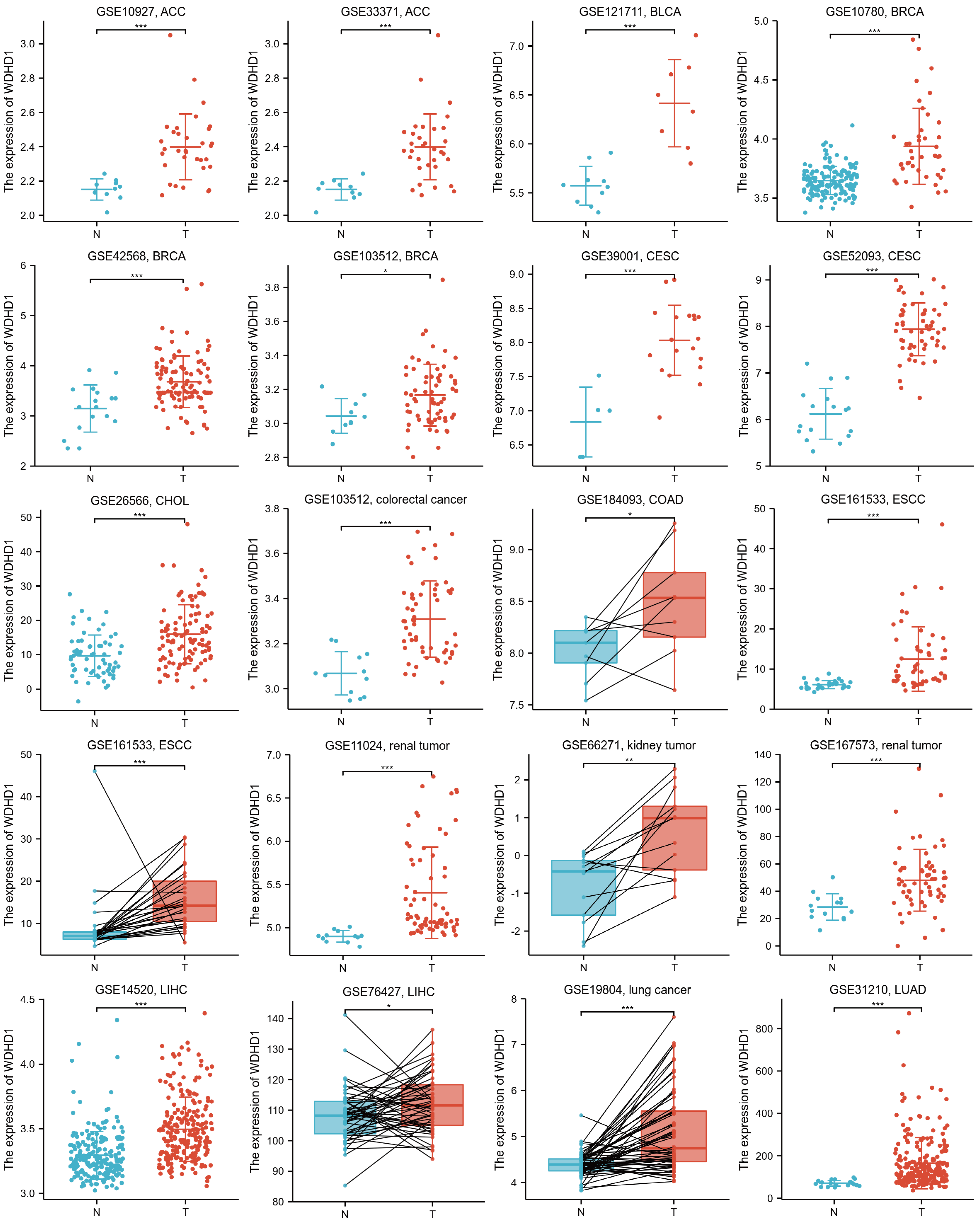

Supplement: Supplementary file 1 — Additional file 1: Figure S1. WDHD1 mRNA expression between tumor and normal tissues in 20 independent cohorts from the GEO database. T is short for tumor tissues, and N is short for normal tissues (* p < 0.05, ** p < 0.01, *** p < 0.001). Figure S2. WDHD1 mRNA expression between tumor and normal tissues in additional 22 independent cohorts from the GEO database (* p < 0.05, ** p < 0.01, *** p < 0.001). Figure S3. WDHD1 protein expression between normal and tumor tissues by the UALCAN (**** p < 0.0001, ns, not statistically significant). Figure S4. The ROC curves indicate that WDHD1 has an excellent diagnostic value in the TCGA pan-cancer cohort. The true positive rate (TPR) is shown on the Y-axis and the false positive rate (FPR) is shown on the X-axis. Diagnostic accuracy increases with a larger area under the curve (AUC). Figure S5. The diagnostic value of WDHD1 was evaluated using the GEO dataset (41 independent cohorts in total) as external validation. Figure S6. The relationship between WDHD1 and disease-specific survival (DSS). (A) A DSS forest plot of the pan-cancer cohort. Tumors are arranged according to different origins of tissue (color distinction). The association between WDHD1 expression and patient DSS in KIRP (B), BLCA (C), LIHC (D), PAAD (E), LGG (F), LUAD (G), ACC (H), MESO (I), SARC (J), and SKCM (K) is analyzed using Kaplan-Meier methods. Figure S7. The relationship between WDHD1 and progression-free interval (PFI). (A) A PFI forest plot of the pan-cancer cohort. The association between WDHD1 expression and patient PFI in KICH (B), PRAD (C), BLCA (D), OV (E), PAAD (F), LIHC (G), LGG (H), GBM (I), LUAD (J), ACC (K), PCPG (L), MESO (M), and SARC (N) is analyzed using Kaplan-Meier methods. Figure S8. WDHD1 survival analysis using 26 independent cohorts from the GEO datasets. In most cases, patient with high WDHD1 expression has a significant worse prognosis. Figure S9. Survival analysis of WDHD1 from the PrognoScan database. A total of 16 independe [file 12957_2023_3187_MOESM1_ESM.zip › Additional file 1/FigureS1.tif]

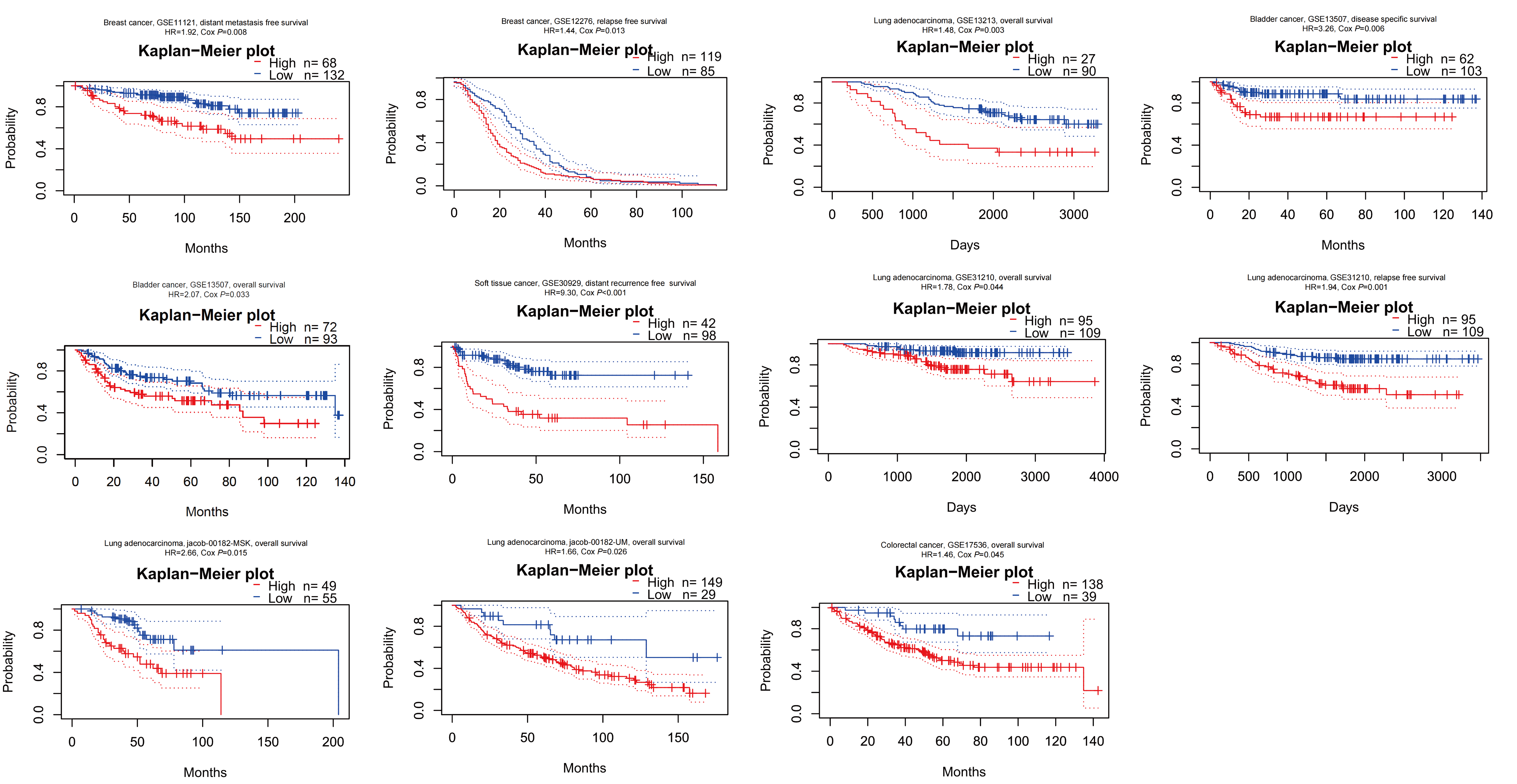

Supplement: Supplementary file 1 — Additional file 1: Figure S1. WDHD1 mRNA expression between tumor and normal tissues in 20 independent cohorts from the GEO database. T is short for tumor tissues, and N is short for normal tissues (* p < 0.05, ** p < 0.01, *** p < 0.001). Figure S2. WDHD1 mRNA expression between tumor and normal tissues in additional 22 independent cohorts from the GEO database (* p < 0.05, ** p < 0.01, *** p < 0.001). Figure S3. WDHD1 protein expression between normal and tumor tissues by the UALCAN (**** p < 0.0001, ns, not statistically significant). Figure S4. The ROC curves indicate that WDHD1 has an excellent diagnostic value in the TCGA pan-cancer cohort. The true positive rate (TPR) is shown on the Y-axis and the false positive rate (FPR) is shown on the X-axis. Diagnostic accuracy increases with a larger area under the curve (AUC). Figure S5. The diagnostic value of WDHD1 was evaluated using the GEO dataset (41 independent cohorts in total) as external validation. Figure S6. The relationship between WDHD1 and disease-specific survival (DSS). (A) A DSS forest plot of the pan-cancer cohort. Tumors are arranged according to different origins of tissue (color distinction). The association between WDHD1 expression and patient DSS in KIRP (B), BLCA (C), LIHC (D), PAAD (E), LGG (F), LUAD (G), ACC (H), MESO (I), SARC (J), and SKCM (K) is analyzed using Kaplan-Meier methods. Figure S7. The relationship between WDHD1 and progression-free interval (PFI). (A) A PFI forest plot of the pan-cancer cohort. The association between WDHD1 expression and patient PFI in KICH (B), PRAD (C), BLCA (D), OV (E), PAAD (F), LIHC (G), LGG (H), GBM (I), LUAD (J), ACC (K), PCPG (L), MESO (M), and SARC (N) is analyzed using Kaplan-Meier methods. Figure S8. WDHD1 survival analysis using 26 independent cohorts from the GEO datasets. In most cases, patient with high WDHD1 expression has a significant worse prognosis. Figure S9. Survival analysis of WDHD1 from the PrognoScan database. A total of 16 independe [file 12957_2023_3187_MOESM1_ESM.zip › Additional file 1/FigureS10.tif]

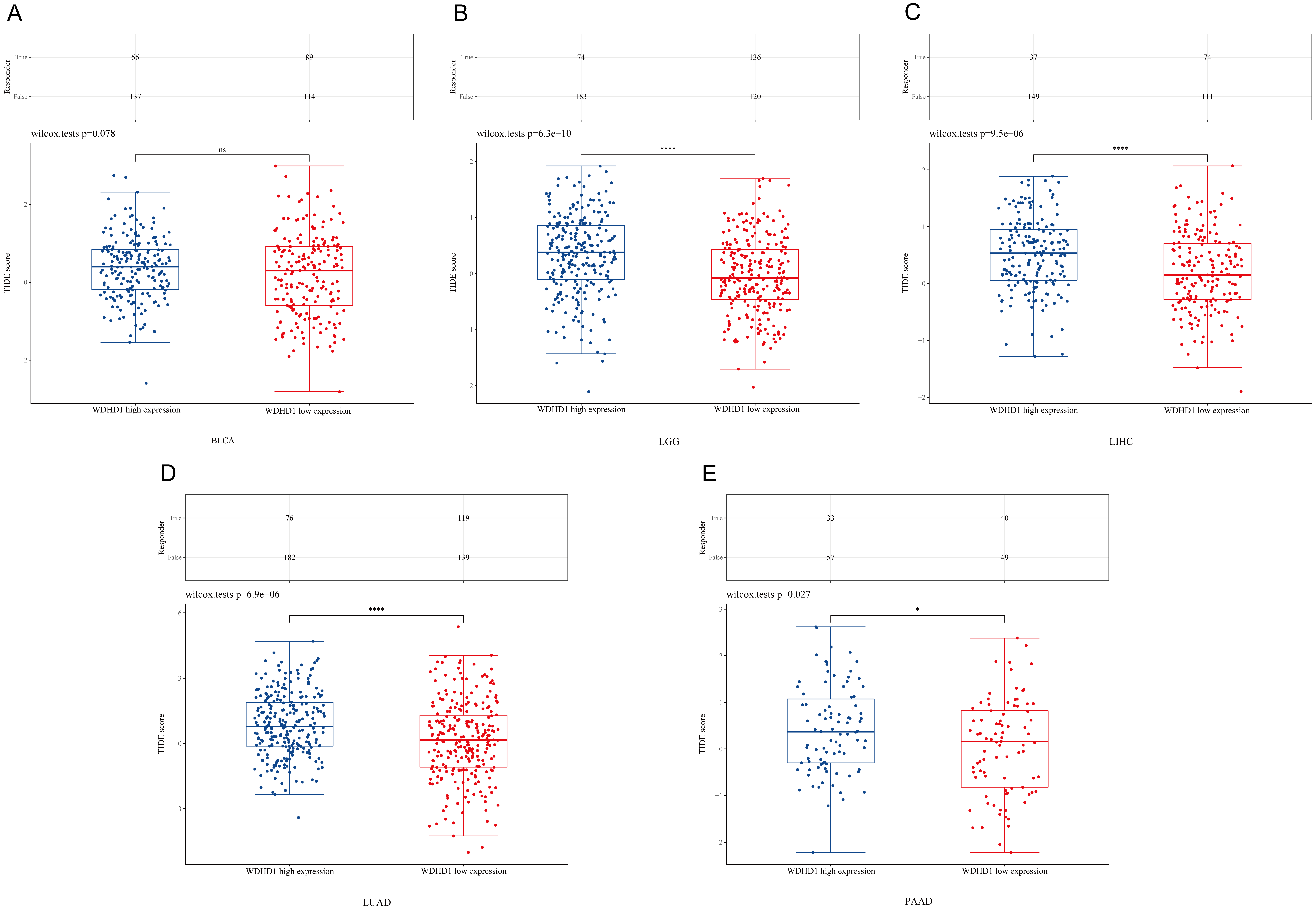

Supplement: Supplementary file 1 — Additional file 1: Figure S1. WDHD1 mRNA expression between tumor and normal tissues in 20 independent cohorts from the GEO database. T is short for tumor tissues, and N is short for normal tissues (* p < 0.05, ** p < 0.01, *** p < 0.001). Figure S2. WDHD1 mRNA expression between tumor and normal tissues in additional 22 independent cohorts from the GEO database (* p < 0.05, ** p < 0.01, *** p < 0.001). Figure S3. WDHD1 protein expression between normal and tumor tissues by the UALCAN (**** p < 0.0001, ns, not statistically significant). Figure S4. The ROC curves indicate that WDHD1 has an excellent diagnostic value in the TCGA pan-cancer cohort. The true positive rate (TPR) is shown on the Y-axis and the false positive rate (FPR) is shown on the X-axis. Diagnostic accuracy increases with a larger area under the curve (AUC). Figure S5. The diagnostic value of WDHD1 was evaluated using the GEO dataset (41 independent cohorts in total) as external validation. Figure S6. The relationship between WDHD1 and disease-specific survival (DSS). (A) A DSS forest plot of the pan-cancer cohort. Tumors are arranged according to different origins of tissue (color distinction). The association between WDHD1 expression and patient DSS in KIRP (B), BLCA (C), LIHC (D), PAAD (E), LGG (F), LUAD (G), ACC (H), MESO (I), SARC (J), and SKCM (K) is analyzed using Kaplan-Meier methods. Figure S7. The relationship between WDHD1 and progression-free interval (PFI). (A) A PFI forest plot of the pan-cancer cohort. The association between WDHD1 expression and patient PFI in KICH (B), PRAD (C), BLCA (D), OV (E), PAAD (F), LIHC (G), LGG (H), GBM (I), LUAD (J), ACC (K), PCPG (L), MESO (M), and SARC (N) is analyzed using Kaplan-Meier methods. Figure S8. WDHD1 survival analysis using 26 independent cohorts from the GEO datasets. In most cases, patient with high WDHD1 expression has a significant worse prognosis. Figure S9. Survival analysis of WDHD1 from the PrognoScan database. A total of 16 independe [file 12957_2023_3187_MOESM1_ESM.zip › Additional file 1/FigureS11.tif]

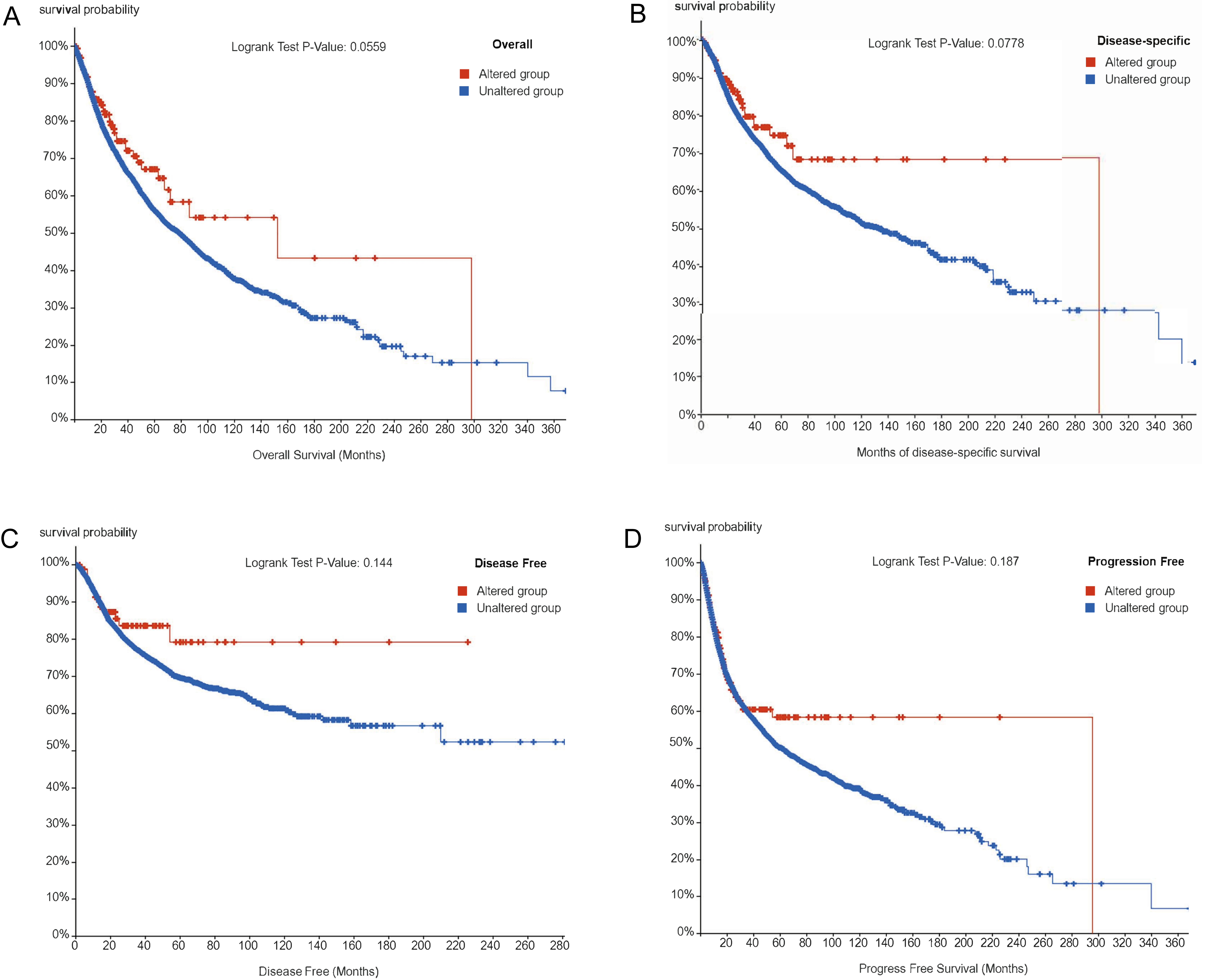

Supplement: Supplementary file 1 — Additional file 1: Figure S1. WDHD1 mRNA expression between tumor and normal tissues in 20 independent cohorts from the GEO database. T is short for tumor tissues, and N is short for normal tissues (* p < 0.05, ** p < 0.01, *** p < 0.001). Figure S2. WDHD1 mRNA expression between tumor and normal tissues in additional 22 independent cohorts from the GEO database (* p < 0.05, ** p < 0.01, *** p < 0.001). Figure S3. WDHD1 protein expression between normal and tumor tissues by the UALCAN (**** p < 0.0001, ns, not statistically significant). Figure S4. The ROC curves indicate that WDHD1 has an excellent diagnostic value in the TCGA pan-cancer cohort. The true positive rate (TPR) is shown on the Y-axis and the false positive rate (FPR) is shown on the X-axis. Diagnostic accuracy increases with a larger area under the curve (AUC). Figure S5. The diagnostic value of WDHD1 was evaluated using the GEO dataset (41 independent cohorts in total) as external validation. Figure S6. The relationship between WDHD1 and disease-specific survival (DSS). (A) A DSS forest plot of the pan-cancer cohort. Tumors are arranged according to different origins of tissue (color distinction). The association between WDHD1 expression and patient DSS in KIRP (B), BLCA (C), LIHC (D), PAAD (E), LGG (F), LUAD (G), ACC (H), MESO (I), SARC (J), and SKCM (K) is analyzed using Kaplan-Meier methods. Figure S7. The relationship between WDHD1 and progression-free interval (PFI). (A) A PFI forest plot of the pan-cancer cohort. The association between WDHD1 expression and patient PFI in KICH (B), PRAD (C), BLCA (D), OV (E), PAAD (F), LIHC (G), LGG (H), GBM (I), LUAD (J), ACC (K), PCPG (L), MESO (M), and SARC (N) is analyzed using Kaplan-Meier methods. Figure S8. WDHD1 survival analysis using 26 independent cohorts from the GEO datasets. In most cases, patient with high WDHD1 expression has a significant worse prognosis. Figure S9. Survival analysis of WDHD1 from the PrognoScan database. A total of 16 independe [file 12957_2023_3187_MOESM1_ESM.zip › Additional file 1/FigureS12.tif]

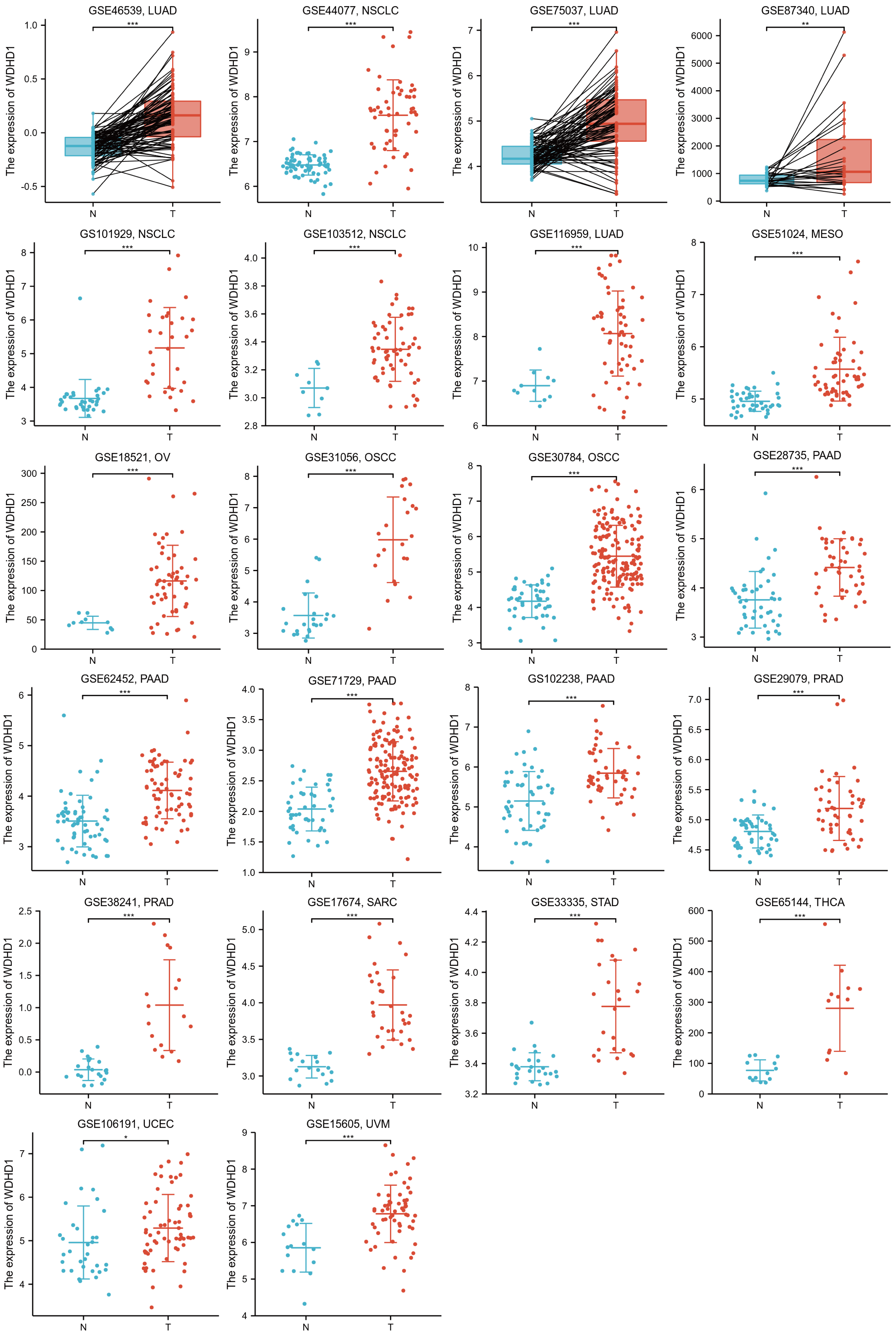

Supplement: Supplementary file 1 — Additional file 1: Figure S1. WDHD1 mRNA expression between tumor and normal tissues in 20 independent cohorts from the GEO database. T is short for tumor tissues, and N is short for normal tissues (* p < 0.05, ** p < 0.01, *** p < 0.001). Figure S2. WDHD1 mRNA expression between tumor and normal tissues in additional 22 independent cohorts from the GEO database (* p < 0.05, ** p < 0.01, *** p < 0.001). Figure S3. WDHD1 protein expression between normal and tumor tissues by the UALCAN (**** p < 0.0001, ns, not statistically significant). Figure S4. The ROC curves indicate that WDHD1 has an excellent diagnostic value in the TCGA pan-cancer cohort. The true positive rate (TPR) is shown on the Y-axis and the false positive rate (FPR) is shown on the X-axis. Diagnostic accuracy increases with a larger area under the curve (AUC). Figure S5. The diagnostic value of WDHD1 was evaluated using the GEO dataset (41 independent cohorts in total) as external validation. Figure S6. The relationship between WDHD1 and disease-specific survival (DSS). (A) A DSS forest plot of the pan-cancer cohort. Tumors are arranged according to different origins of tissue (color distinction). The association between WDHD1 expression and patient DSS in KIRP (B), BLCA (C), LIHC (D), PAAD (E), LGG (F), LUAD (G), ACC (H), MESO (I), SARC (J), and SKCM (K) is analyzed using Kaplan-Meier methods. Figure S7. The relationship between WDHD1 and progression-free interval (PFI). (A) A PFI forest plot of the pan-cancer cohort. The association between WDHD1 expression and patient PFI in KICH (B), PRAD (C), BLCA (D), OV (E), PAAD (F), LIHC (G), LGG (H), GBM (I), LUAD (J), ACC (K), PCPG (L), MESO (M), and SARC (N) is analyzed using Kaplan-Meier methods. Figure S8. WDHD1 survival analysis using 26 independent cohorts from the GEO datasets. In most cases, patient with high WDHD1 expression has a significant worse prognosis. Figure S9. Survival analysis of WDHD1 from the PrognoScan database. A total of 16 independe [file 12957_2023_3187_MOESM1_ESM.zip › Additional file 1/FigureS2.tif]

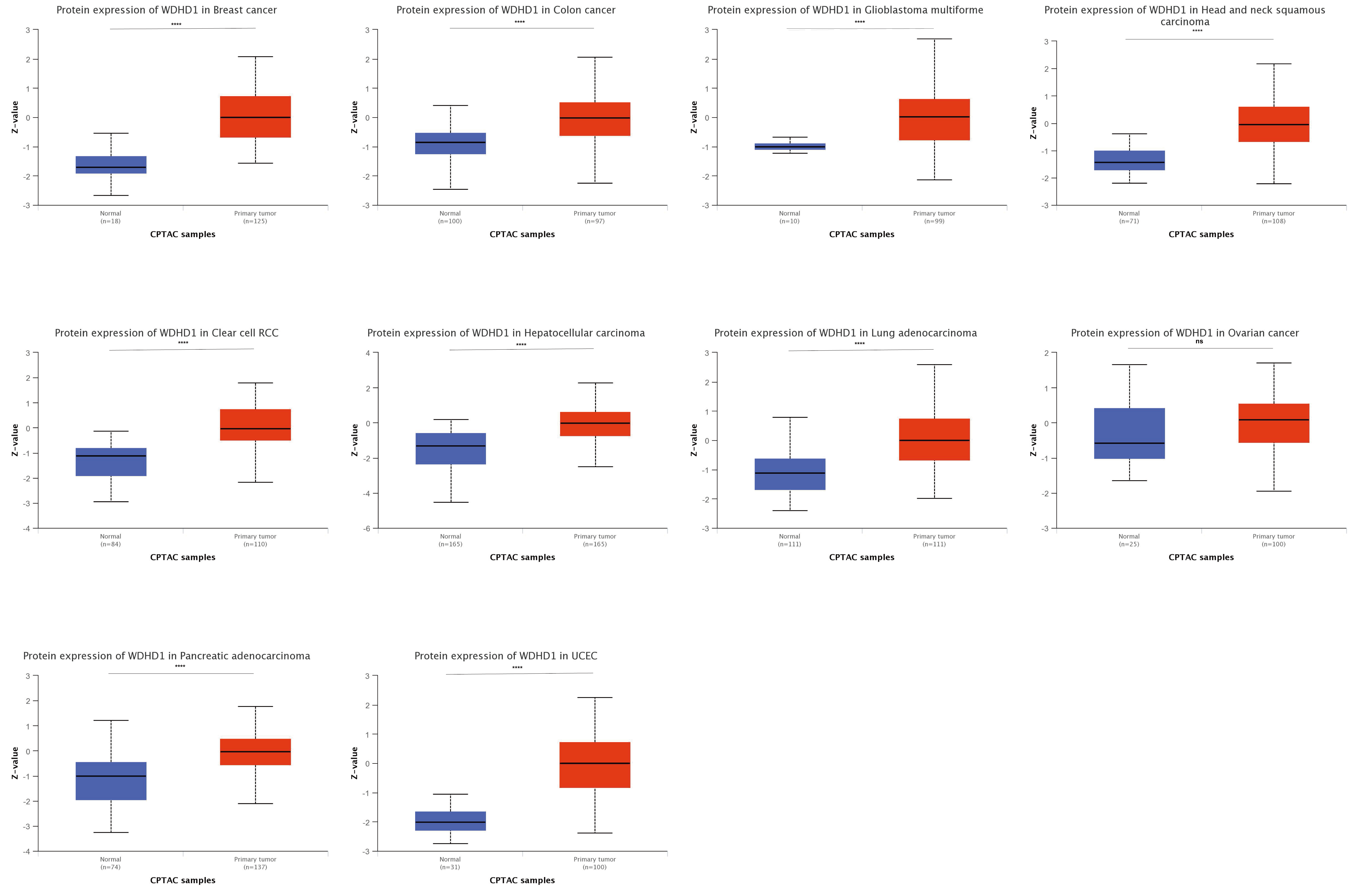

Supplement: Supplementary file 1 — Additional file 1: Figure S1. WDHD1 mRNA expression between tumor and normal tissues in 20 independent cohorts from the GEO database. T is short for tumor tissues, and N is short for normal tissues (* p < 0.05, ** p < 0.01, *** p < 0.001). Figure S2. WDHD1 mRNA expression between tumor and normal tissues in additional 22 independent cohorts from the GEO database (* p < 0.05, ** p < 0.01, *** p < 0.001). Figure S3. WDHD1 protein expression between normal and tumor tissues by the UALCAN (**** p < 0.0001, ns, not statistically significant). Figure S4. The ROC curves indicate that WDHD1 has an excellent diagnostic value in the TCGA pan-cancer cohort. The true positive rate (TPR) is shown on the Y-axis and the false positive rate (FPR) is shown on the X-axis. Diagnostic accuracy increases with a larger area under the curve (AUC). Figure S5. The diagnostic value of WDHD1 was evaluated using the GEO dataset (41 independent cohorts in total) as external validation. Figure S6. The relationship between WDHD1 and disease-specific survival (DSS). (A) A DSS forest plot of the pan-cancer cohort. Tumors are arranged according to different origins of tissue (color distinction). The association between WDHD1 expression and patient DSS in KIRP (B), BLCA (C), LIHC (D), PAAD (E), LGG (F), LUAD (G), ACC (H), MESO (I), SARC (J), and SKCM (K) is analyzed using Kaplan-Meier methods. Figure S7. The relationship between WDHD1 and progression-free interval (PFI). (A) A PFI forest plot of the pan-cancer cohort. The association between WDHD1 expression and patient PFI in KICH (B), PRAD (C), BLCA (D), OV (E), PAAD (F), LIHC (G), LGG (H), GBM (I), LUAD (J), ACC (K), PCPG (L), MESO (M), and SARC (N) is analyzed using Kaplan-Meier methods. Figure S8. WDHD1 survival analysis using 26 independent cohorts from the GEO datasets. In most cases, patient with high WDHD1 expression has a significant worse prognosis. Figure S9. Survival analysis of WDHD1 from the PrognoScan database. A total of 16 independe [file 12957_2023_3187_MOESM1_ESM.zip › Additional file 1/FigureS3.tif]

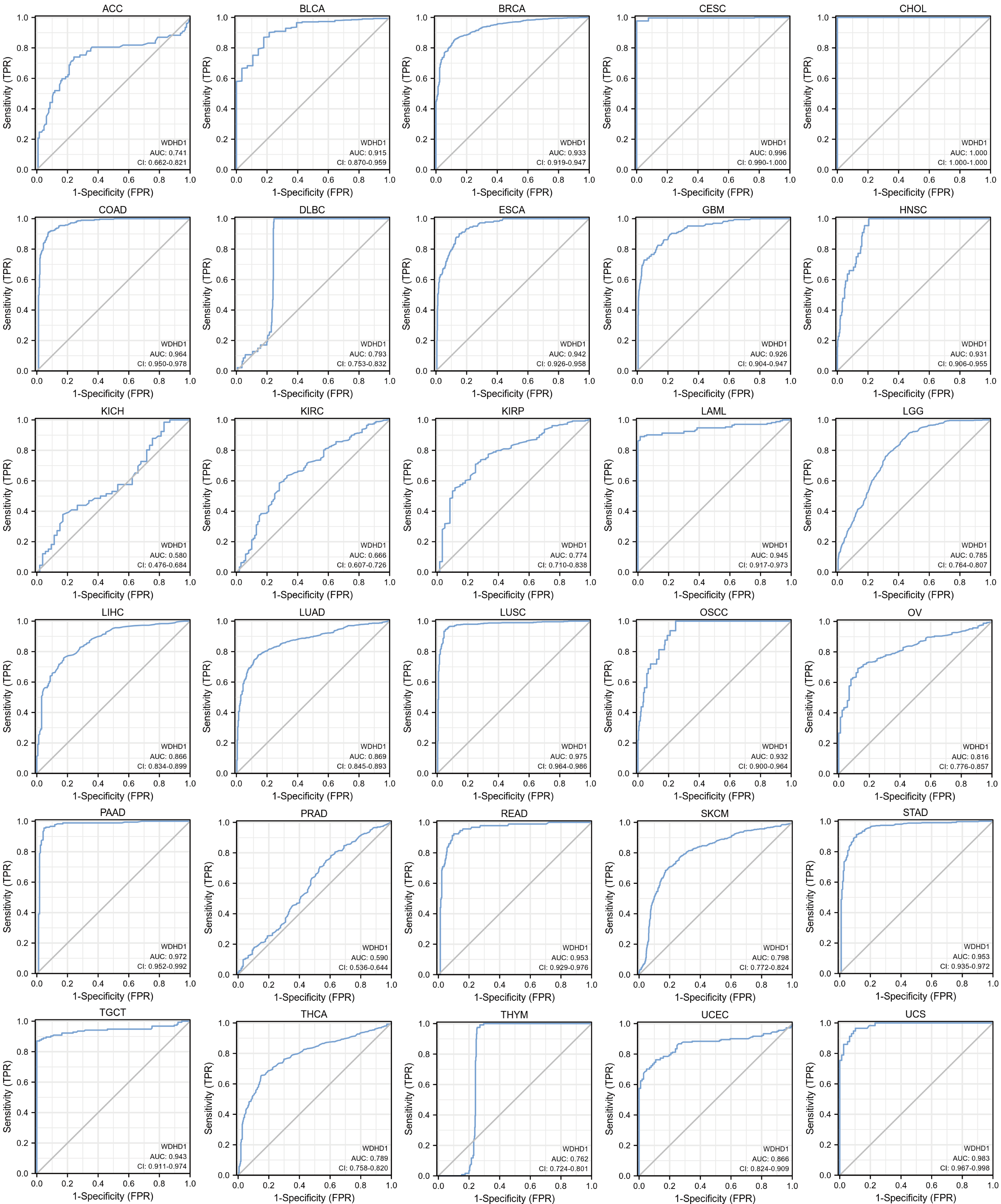

Supplement: Supplementary file 1 — Additional file 1: Figure S1. WDHD1 mRNA expression between tumor and normal tissues in 20 independent cohorts from the GEO database. T is short for tumor tissues, and N is short for normal tissues (* p < 0.05, ** p < 0.01, *** p < 0.001). Figure S2. WDHD1 mRNA expression between tumor and normal tissues in additional 22 independent cohorts from the GEO database (* p < 0.05, ** p < 0.01, *** p < 0.001). Figure S3. WDHD1 protein expression between normal and tumor tissues by the UALCAN (**** p < 0.0001, ns, not statistically significant). Figure S4. The ROC curves indicate that WDHD1 has an excellent diagnostic value in the TCGA pan-cancer cohort. The true positive rate (TPR) is shown on the Y-axis and the false positive rate (FPR) is shown on the X-axis. Diagnostic accuracy increases with a larger area under the curve (AUC). Figure S5. The diagnostic value of WDHD1 was evaluated using the GEO dataset (41 independent cohorts in total) as external validation. Figure S6. The relationship between WDHD1 and disease-specific survival (DSS). (A) A DSS forest plot of the pan-cancer cohort. Tumors are arranged according to different origins of tissue (color distinction). The association between WDHD1 expression and patient DSS in KIRP (B), BLCA (C), LIHC (D), PAAD (E), LGG (F), LUAD (G), ACC (H), MESO (I), SARC (J), and SKCM (K) is analyzed using Kaplan-Meier methods. Figure S7. The relationship between WDHD1 and progression-free interval (PFI). (A) A PFI forest plot of the pan-cancer cohort. The association between WDHD1 expression and patient PFI in KICH (B), PRAD (C), BLCA (D), OV (E), PAAD (F), LIHC (G), LGG (H), GBM (I), LUAD (J), ACC (K), PCPG (L), MESO (M), and SARC (N) is analyzed using Kaplan-Meier methods. Figure S8. WDHD1 survival analysis using 26 independent cohorts from the GEO datasets. In most cases, patient with high WDHD1 expression has a significant worse prognosis. Figure S9. Survival analysis of WDHD1 from the PrognoScan database. A total of 16 independe [file 12957_2023_3187_MOESM1_ESM.zip › Additional file 1/FigureS4.tif]

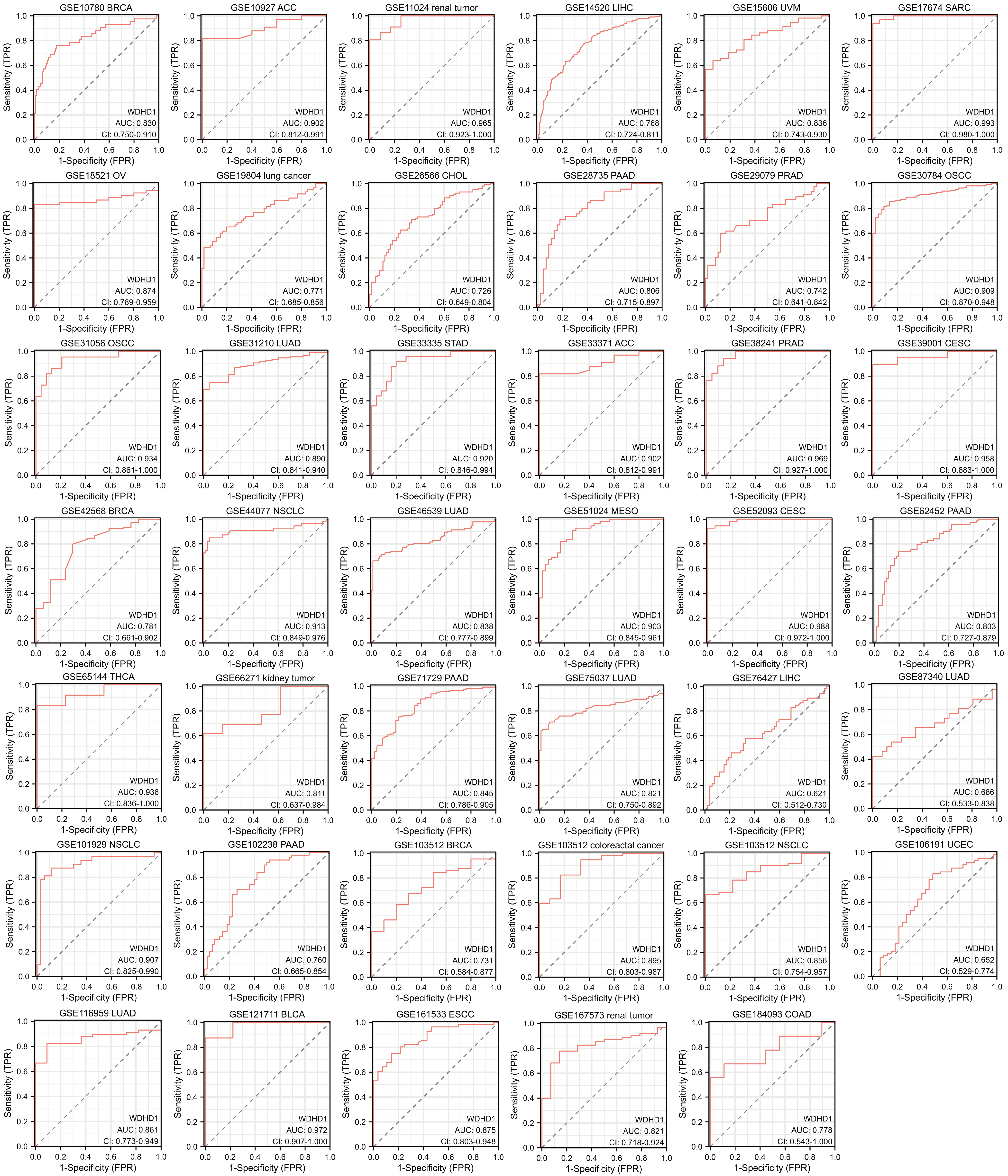

Supplement: Supplementary file 1 — Additional file 1: Figure S1. WDHD1 mRNA expression between tumor and normal tissues in 20 independent cohorts from the GEO database. T is short for tumor tissues, and N is short for normal tissues (* p < 0.05, ** p < 0.01, *** p < 0.001). Figure S2. WDHD1 mRNA expression between tumor and normal tissues in additional 22 independent cohorts from the GEO database (* p < 0.05, ** p < 0.01, *** p < 0.001). Figure S3. WDHD1 protein expression between normal and tumor tissues by the UALCAN (**** p < 0.0001, ns, not statistically significant). Figure S4. The ROC curves indicate that WDHD1 has an excellent diagnostic value in the TCGA pan-cancer cohort. The true positive rate (TPR) is shown on the Y-axis and the false positive rate (FPR) is shown on the X-axis. Diagnostic accuracy increases with a larger area under the curve (AUC). Figure S5. The diagnostic value of WDHD1 was evaluated using the GEO dataset (41 independent cohorts in total) as external validation. Figure S6. The relationship between WDHD1 and disease-specific survival (DSS). (A) A DSS forest plot of the pan-cancer cohort. Tumors are arranged according to different origins of tissue (color distinction). The association between WDHD1 expression and patient DSS in KIRP (B), BLCA (C), LIHC (D), PAAD (E), LGG (F), LUAD (G), ACC (H), MESO (I), SARC (J), and SKCM (K) is analyzed using Kaplan-Meier methods. Figure S7. The relationship between WDHD1 and progression-free interval (PFI). (A) A PFI forest plot of the pan-cancer cohort. The association between WDHD1 expression and patient PFI in KICH (B), PRAD (C), BLCA (D), OV (E), PAAD (F), LIHC (G), LGG (H), GBM (I), LUAD (J), ACC (K), PCPG (L), MESO (M), and SARC (N) is analyzed using Kaplan-Meier methods. Figure S8. WDHD1 survival analysis using 26 independent cohorts from the GEO datasets. In most cases, patient with high WDHD1 expression has a significant worse prognosis. Figure S9. Survival analysis of WDHD1 from the PrognoScan database. A total of 16 independe [file 12957_2023_3187_MOESM1_ESM.zip › Additional file 1/FigureS5.tif]

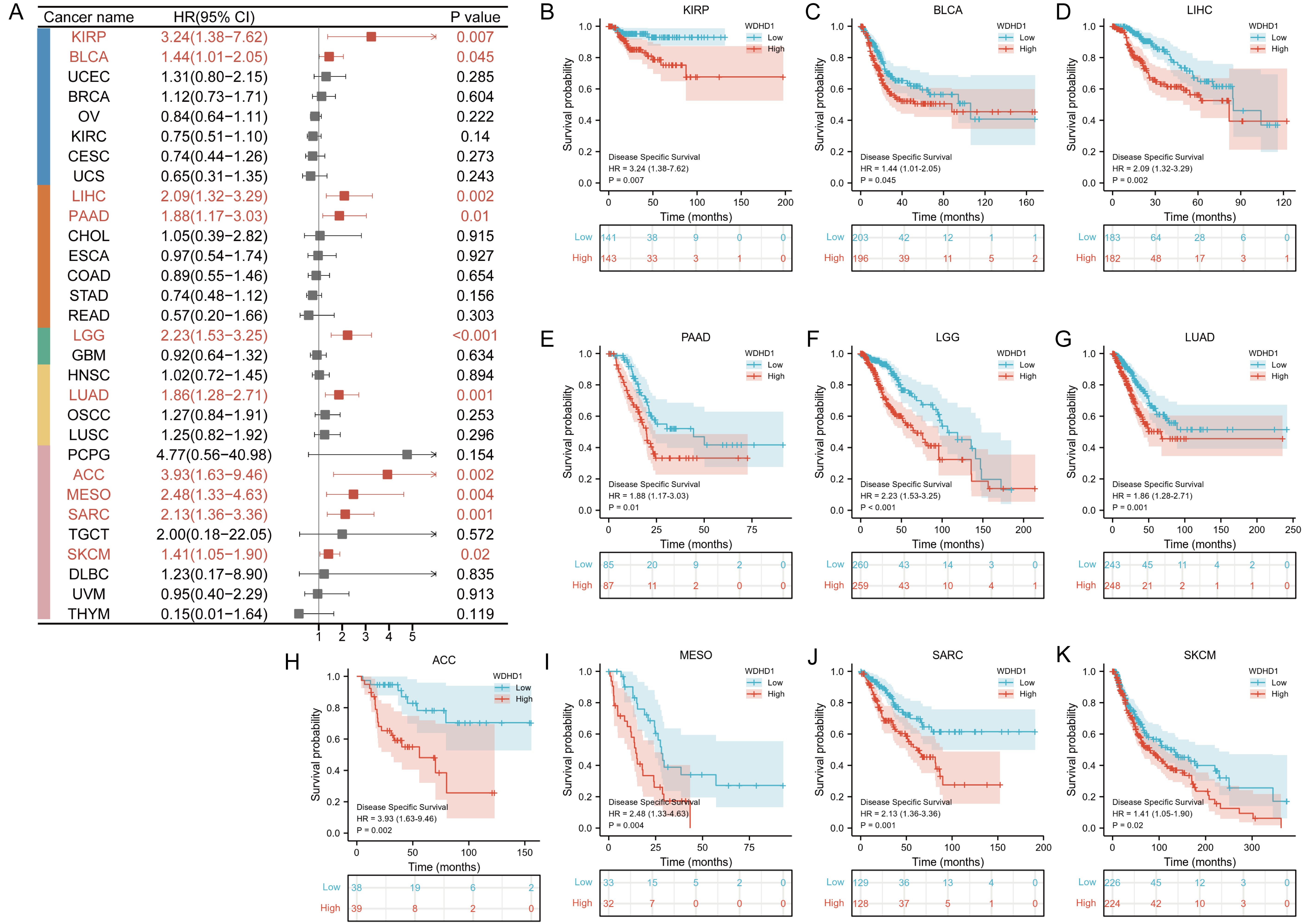

Supplement: Supplementary file 1 — Additional file 1: Figure S1. WDHD1 mRNA expression between tumor and normal tissues in 20 independent cohorts from the GEO database. T is short for tumor tissues, and N is short for normal tissues (* p < 0.05, ** p < 0.01, *** p < 0.001). Figure S2. WDHD1 mRNA expression between tumor and normal tissues in additional 22 independent cohorts from the GEO database (* p < 0.05, ** p < 0.01, *** p < 0.001). Figure S3. WDHD1 protein expression between normal and tumor tissues by the UALCAN (**** p < 0.0001, ns, not statistically significant). Figure S4. The ROC curves indicate that WDHD1 has an excellent diagnostic value in the TCGA pan-cancer cohort. The true positive rate (TPR) is shown on the Y-axis and the false positive rate (FPR) is shown on the X-axis. Diagnostic accuracy increases with a larger area under the curve (AUC). Figure S5. The diagnostic value of WDHD1 was evaluated using the GEO dataset (41 independent cohorts in total) as external validation. Figure S6. The relationship between WDHD1 and disease-specific survival (DSS). (A) A DSS forest plot of the pan-cancer cohort. Tumors are arranged according to different origins of tissue (color distinction). The association between WDHD1 expression and patient DSS in KIRP (B), BLCA (C), LIHC (D), PAAD (E), LGG (F), LUAD (G), ACC (H), MESO (I), SARC (J), and SKCM (K) is analyzed using Kaplan-Meier methods. Figure S7. The relationship between WDHD1 and progression-free interval (PFI). (A) A PFI forest plot of the pan-cancer cohort. The association between WDHD1 expression and patient PFI in KICH (B), PRAD (C), BLCA (D), OV (E), PAAD (F), LIHC (G), LGG (H), GBM (I), LUAD (J), ACC (K), PCPG (L), MESO (M), and SARC (N) is analyzed using Kaplan-Meier methods. Figure S8. WDHD1 survival analysis using 26 independent cohorts from the GEO datasets. In most cases, patient with high WDHD1 expression has a significant worse prognosis. Figure S9. Survival analysis of WDHD1 from the PrognoScan database. A total of 16 independe [file 12957_2023_3187_MOESM1_ESM.zip › Additional file 1/FigureS6.tif]

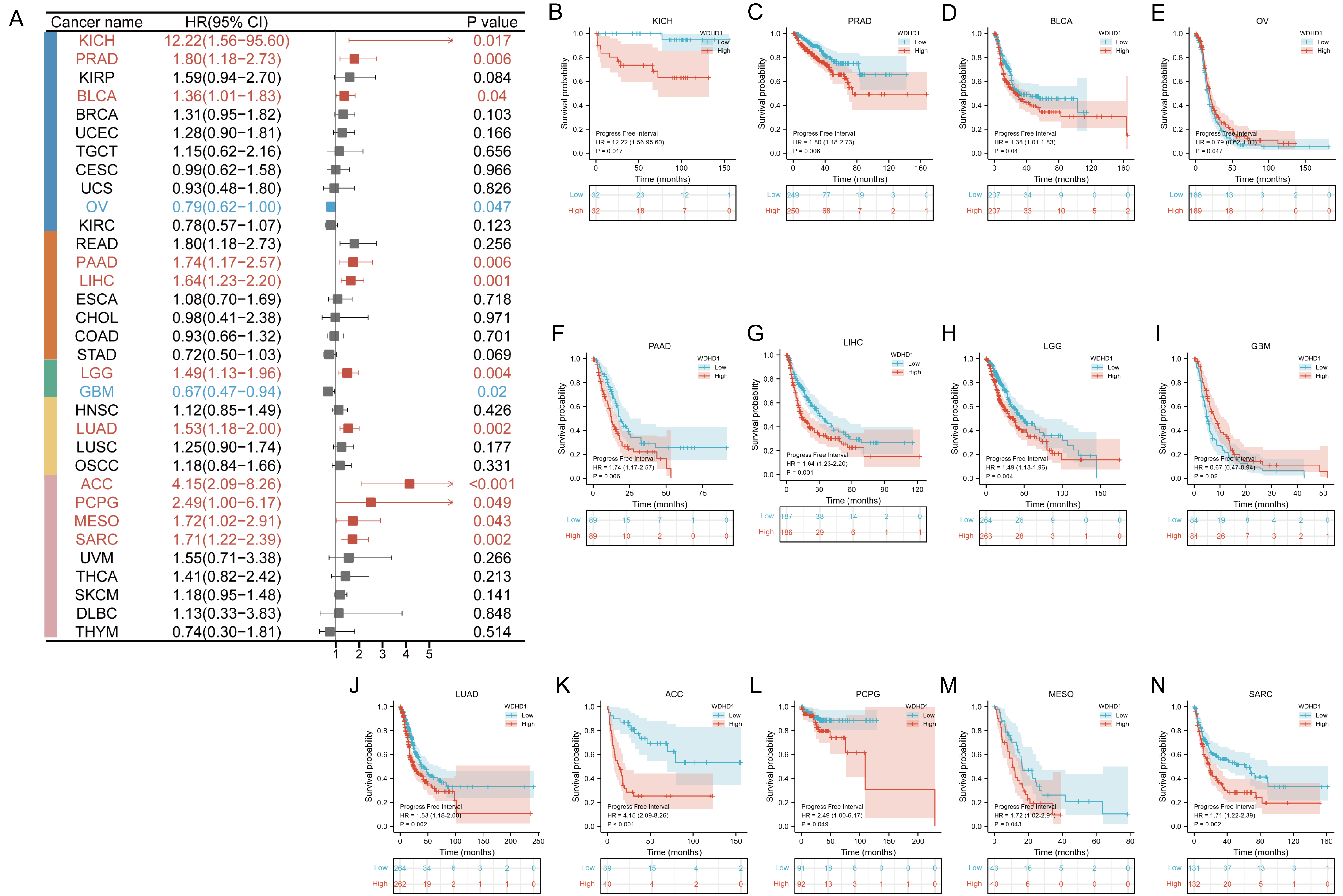

Supplement: Supplementary file 1 — Additional file 1: Figure S1. WDHD1 mRNA expression between tumor and normal tissues in 20 independent cohorts from the GEO database. T is short for tumor tissues, and N is short for normal tissues (* p < 0.05, ** p < 0.01, *** p < 0.001). Figure S2. WDHD1 mRNA expression between tumor and normal tissues in additional 22 independent cohorts from the GEO database (* p < 0.05, ** p < 0.01, *** p < 0.001). Figure S3. WDHD1 protein expression between normal and tumor tissues by the UALCAN (**** p < 0.0001, ns, not statistically significant). Figure S4. The ROC curves indicate that WDHD1 has an excellent diagnostic value in the TCGA pan-cancer cohort. The true positive rate (TPR) is shown on the Y-axis and the false positive rate (FPR) is shown on the X-axis. Diagnostic accuracy increases with a larger area under the curve (AUC). Figure S5. The diagnostic value of WDHD1 was evaluated using the GEO dataset (41 independent cohorts in total) as external validation. Figure S6. The relationship between WDHD1 and disease-specific survival (DSS). (A) A DSS forest plot of the pan-cancer cohort. Tumors are arranged according to different origins of tissue (color distinction). The association between WDHD1 expression and patient DSS in KIRP (B), BLCA (C), LIHC (D), PAAD (E), LGG (F), LUAD (G), ACC (H), MESO (I), SARC (J), and SKCM (K) is analyzed using Kaplan-Meier methods. Figure S7. The relationship between WDHD1 and progression-free interval (PFI). (A) A PFI forest plot of the pan-cancer cohort. The association between WDHD1 expression and patient PFI in KICH (B), PRAD (C), BLCA (D), OV (E), PAAD (F), LIHC (G), LGG (H), GBM (I), LUAD (J), ACC (K), PCPG (L), MESO (M), and SARC (N) is analyzed using Kaplan-Meier methods. Figure S8. WDHD1 survival analysis using 26 independent cohorts from the GEO datasets. In most cases, patient with high WDHD1 expression has a significant worse prognosis. Figure S9. Survival analysis of WDHD1 from the PrognoScan database. A total of 16 independe [file 12957_2023_3187_MOESM1_ESM.zip › Additional file 1/FigureS7.tif]

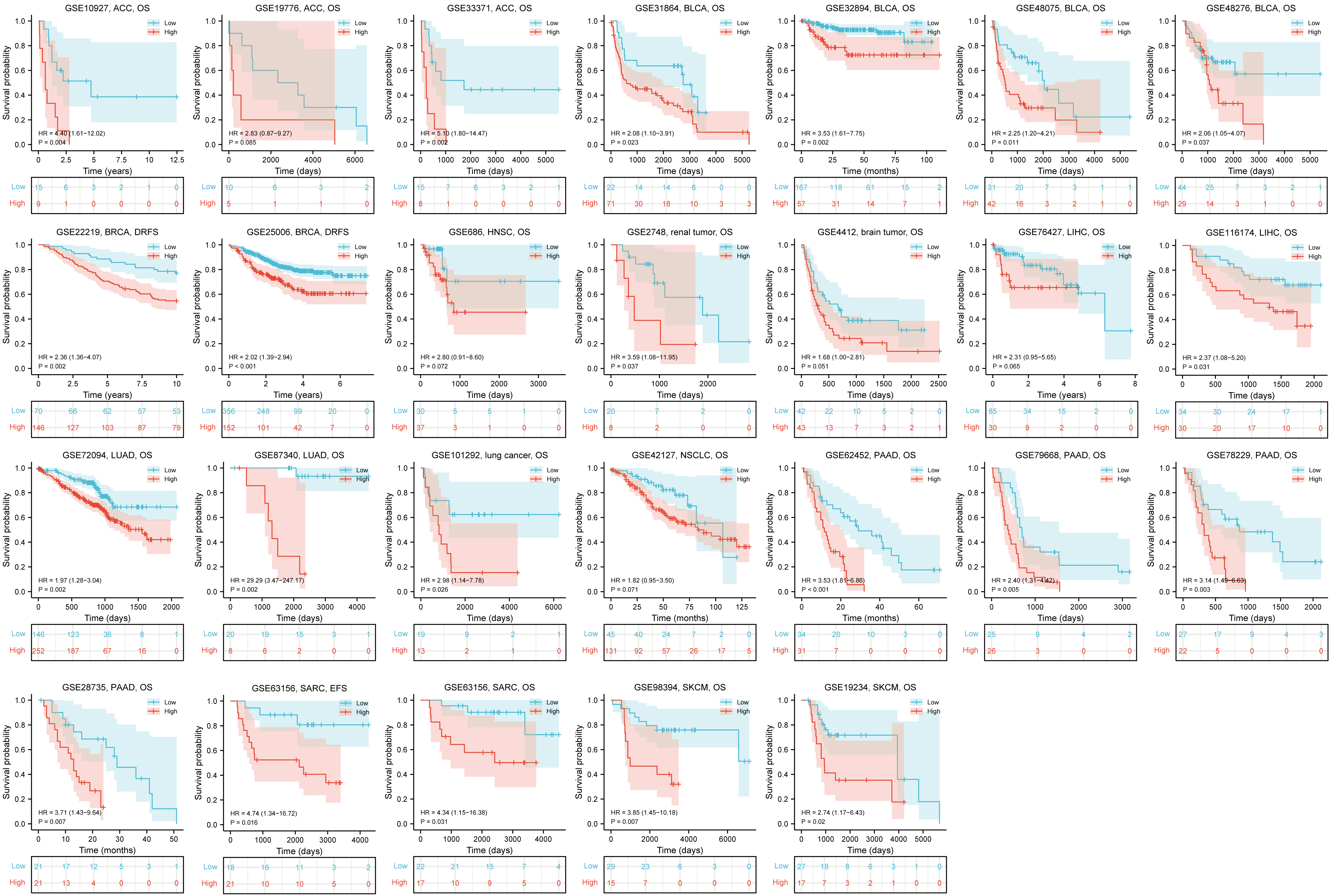

Supplement: Supplementary file 1 — Additional file 1: Figure S1. WDHD1 mRNA expression between tumor and normal tissues in 20 independent cohorts from the GEO database. T is short for tumor tissues, and N is short for normal tissues (* p < 0.05, ** p < 0.01, *** p < 0.001). Figure S2. WDHD1 mRNA expression between tumor and normal tissues in additional 22 independent cohorts from the GEO database (* p < 0.05, ** p < 0.01, *** p < 0.001). Figure S3. WDHD1 protein expression between normal and tumor tissues by the UALCAN (**** p < 0.0001, ns, not statistically significant). Figure S4. The ROC curves indicate that WDHD1 has an excellent diagnostic value in the TCGA pan-cancer cohort. The true positive rate (TPR) is shown on the Y-axis and the false positive rate (FPR) is shown on the X-axis. Diagnostic accuracy increases with a larger area under the curve (AUC). Figure S5. The diagnostic value of WDHD1 was evaluated using the GEO dataset (41 independent cohorts in total) as external validation. Figure S6. The relationship between WDHD1 and disease-specific survival (DSS). (A) A DSS forest plot of the pan-cancer cohort. Tumors are arranged according to different origins of tissue (color distinction). The association between WDHD1 expression and patient DSS in KIRP (B), BLCA (C), LIHC (D), PAAD (E), LGG (F), LUAD (G), ACC (H), MESO (I), SARC (J), and SKCM (K) is analyzed using Kaplan-Meier methods. Figure S7. The relationship between WDHD1 and progression-free interval (PFI). (A) A PFI forest plot of the pan-cancer cohort. The association between WDHD1 expression and patient PFI in KICH (B), PRAD (C), BLCA (D), OV (E), PAAD (F), LIHC (G), LGG (H), GBM (I), LUAD (J), ACC (K), PCPG (L), MESO (M), and SARC (N) is analyzed using Kaplan-Meier methods. Figure S8. WDHD1 survival analysis using 26 independent cohorts from the GEO datasets. In most cases, patient with high WDHD1 expression has a significant worse prognosis. Figure S9. Survival analysis of WDHD1 from the PrognoScan database. A total of 16 independe [file 12957_2023_3187_MOESM1_ESM.zip › Additional file 1/FigureS8.tif]

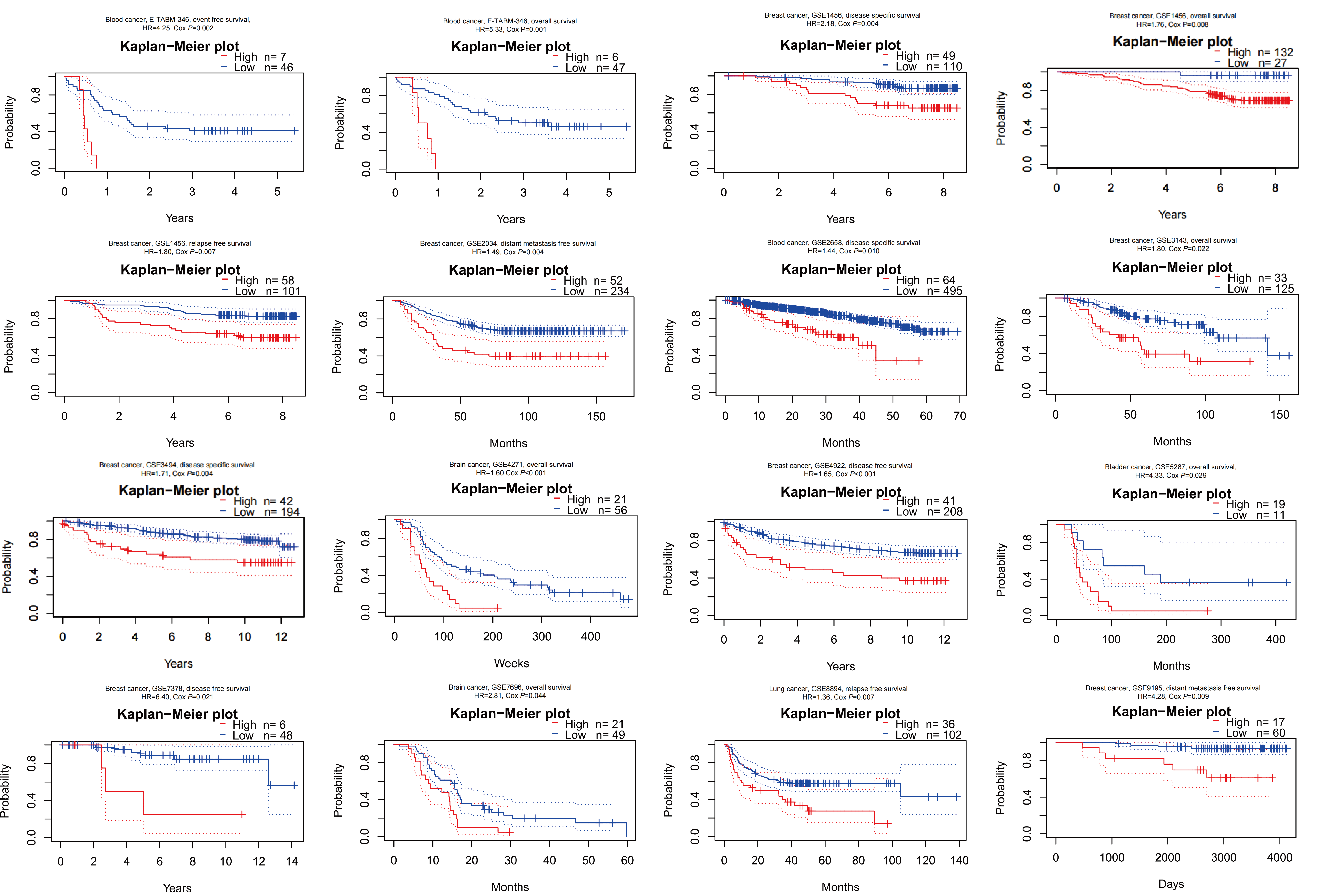

Supplement: Supplementary file 1 — Additional file 1: Figure S1. WDHD1 mRNA expression between tumor and normal tissues in 20 independent cohorts from the GEO database. T is short for tumor tissues, and N is short for normal tissues (* p < 0.05, ** p < 0.01, *** p < 0.001). Figure S2. WDHD1 mRNA expression between tumor and normal tissues in additional 22 independent cohorts from the GEO database (* p < 0.05, ** p < 0.01, *** p < 0.001). Figure S3. WDHD1 protein expression between normal and tumor tissues by the UALCAN (**** p < 0.0001, ns, not statistically significant). Figure S4. The ROC curves indicate that WDHD1 has an excellent diagnostic value in the TCGA pan-cancer cohort. The true positive rate (TPR) is shown on the Y-axis and the false positive rate (FPR) is shown on the X-axis. Diagnostic accuracy increases with a larger area under the curve (AUC). Figure S5. The diagnostic value of WDHD1 was evaluated using the GEO dataset (41 independent cohorts in total) as external validation. Figure S6. The relationship between WDHD1 and disease-specific survival (DSS). (A) A DSS forest plot of the pan-cancer cohort. Tumors are arranged according to different origins of tissue (color distinction). The association between WDHD1 expression and patient DSS in KIRP (B), BLCA (C), LIHC (D), PAAD (E), LGG (F), LUAD (G), ACC (H), MESO (I), SARC (J), and SKCM (K) is analyzed using Kaplan-Meier methods. Figure S7. The relationship between WDHD1 and progression-free interval (PFI). (A) A PFI forest plot of the pan-cancer cohort. The association between WDHD1 expression and patient PFI in KICH (B), PRAD (C), BLCA (D), OV (E), PAAD (F), LIHC (G), LGG (H), GBM (I), LUAD (J), ACC (K), PCPG (L), MESO (M), and SARC (N) is analyzed using Kaplan-Meier methods. Figure S8. WDHD1 survival analysis using 26 independent cohorts from the GEO datasets. In most cases, patient with high WDHD1 expression has a significant worse prognosis. Figure S9. Survival analysis of WDHD1 from the PrognoScan database. A total of 16 independe [file 12957_2023_3187_MOESM1_ESM.zip › Additional file 1/FigureS9.tif]
